# A gain-of-function mutation in *BnaIAA13* disrupts vascular tissue and lateral root development in *Brassica napus*

**DOI:** 10.1093/jxb/erae245

**Published:** 2024-06-02

**Authors:** Jinxiang Gao, Pei Qin, Shan Tang, Liang Guo, Cheng Dai, Jing Wen, Bin Yi, Chaozhi Ma, Jinxiong Shen, Tingdong Fu, Jun Zou, Jinxing Tu

**Affiliations:** National Key Laboratory of Crop Genetic Improvement, College of Plant Science & Technology, Huazhong Agricultural University, Wuhan, 430070, China; College of Agronomy and Biotechnology, Yunnan Agricultural University, Kunming, 650201, China; National Key Laboratory of Crop Genetic Improvement, College of Plant Science & Technology, Huazhong Agricultural University, Wuhan, 430070, China; National Key Laboratory of Crop Genetic Improvement, College of Plant Science & Technology, Huazhong Agricultural University, Wuhan, 430070, China; Yazhouwan National Laboratory, Sanya, Hainan, 572025, China; National Key Laboratory of Crop Genetic Improvement, College of Plant Science & Technology, Huazhong Agricultural University, Wuhan, 430070, China; National Key Laboratory of Crop Genetic Improvement, College of Plant Science & Technology, Huazhong Agricultural University, Wuhan, 430070, China; National Key Laboratory of Crop Genetic Improvement, College of Plant Science & Technology, Huazhong Agricultural University, Wuhan, 430070, China; National Key Laboratory of Crop Genetic Improvement, College of Plant Science & Technology, Huazhong Agricultural University, Wuhan, 430070, China; National Key Laboratory of Crop Genetic Improvement, College of Plant Science & Technology, Huazhong Agricultural University, Wuhan, 430070, China; National Key Laboratory of Crop Genetic Improvement, College of Plant Science & Technology, Huazhong Agricultural University, Wuhan, 430070, China; National Key Laboratory of Crop Genetic Improvement, College of Plant Science & Technology, Huazhong Agricultural University, Wuhan, 430070, China; National Key Laboratory of Crop Genetic Improvement, College of Plant Science & Technology, Huazhong Agricultural University, Wuhan, 430070, China; University of Sydney, Australia

**Keywords:** *BnaA03.IAA13*, *Brassica napus*, lateral root, map-based cloning, plant height, vascular tissue

## Abstract

Rapeseed (*Brassica napus*) is an important oilseed crop worldwide. Plant vascular tissues are responsible for long-distance transport of water and nutrients and for providing mechanical support. The lateral roots absorb water and nutrients. The genetic basis of vascular tissue and lateral root development in rapeseed remains unknown. This study characterized an ethyl methanesulfonate-mutagenized rapeseed mutant, *T16*, which showed dwarf stature, reduced lateral roots, and leaf wilting. SEM observations showed that the internode cells were shortened. Observations of tissue sections revealed defects in vascular bundle development in the stems and petioles. Genetic analysis revealed that the phenotypes of *T16* were controlled by a single semi-dominant nuclear gene. Map-based cloning and genetic complementarity identified *BnaA03.IAA13* as the functional gene; a G-to-A mutation in the second exon changed glycine at position 79 to glutamic acid, disrupting the conserved degron motif VGWPP. Transcriptome analysis in roots and stems showed that auxin and cytokinin signaling pathways were disordered in *T16*. Evolutionary analysis showed that AUXIN/INDOLE-3-ACETIC ACID is conserved during plant evolution. The heterozygote of *T16* showed significantly reduced plant height while maintaining other agronomic traits. Our findings provide novel insights into the regulatory mechanisms of vascular tissue and lateral root development, and offer a new germplasm resource for rapeseed breeding.

## Teaser

*BnaIAA13* can simultaneously regulate the development of vascular tissues and lateral roots in rapeseed; mutations in this gene have potential as a new germplasm in the rapeseed industry.

## Introduction

Vascular tissue is the most important tissue for transporting materials in vascular plants (Sieburth *et al*., 2006). The xylem transports water and minerals to the stems and leaves, and the phloem transports photosynthates and organic metabolites from the source organs to other plant organs (Spicer *et al*., 2010; Sankar *et al*., 2014; Endo *et al*., 2019; Tung *et al*., 2023). Vascular development is regulated by multiple signaling pathways and genes. For example, the small peptide CLAVATA3/ENDOSPERM SURROUNDING REGION41/44 (CLE41/44) promotes the proliferation of vascular stem cells and inhibits the differentiation of procambium into the xylem (Whitford *et al*., 2008). The transcription factor WUSCHEL-RELATED HOMEOBOX 4 (WOX4) promotes cambium proliferation and the development of intrafascicular cambium (Ji *et al*., 2010; Brackmann *et al*., 2018; Smetana *et al*., 2019). Class III HD-ZIP, AUXIN RESPONSE FACTOR5 (ARF5), and CYTOKININ OXIDASE/DEHYDROGENASEs (CKXs) are involved in the development of vascular tissue (Hardtke *et al*., 2004; Mähönen *et al*., 2006; Carlsbecker *et al*., 2010; Köllmer *et al*., 2014). With the development of functional genomic analyses, additional pathways and key genes for this trait have been identified.

The plant root system not only provides mechanical support for the aboveground parts of plants but also plays a crucial role in the absorption and transportation of water and nutrients, as well as the synthesis and storage of organic matter. In contrast to the main root, the lateral root (LR) is a post-embryonic organ that develops from the starting cells of the central sheath of the main root (Casimiro *et al*., 2003). Auxin signaling has a significant effect on LR development. WOX–ARF modules initiate different root types (Zhang *et al*., 2023). LATERAL ORGAN BOUNDARIES DOMAINS (LBDs) regulate LR development, which is directly controlled by ARFs (Okushima *et al*., 2007; Goh *et al*., 2012; Kang *et al*., 2013; Porco *et al*., 2016). Auxin-activated MITOGEN-ACTIVATED PROTEIN KINASE14 (MPK14) positively regulates LR development (Lv *et al*., 2020, 2021). In addition, auxin regulates the phosphorylation of MKK4/5–MPK3/6 through TRANSMEMBRANE KINASE1/4 (TMK1/4) and affects LR development (Huang *et al*., 2019). Cytokinin signaling also plays an important role in LR development. LONELY GUY (LOG) can activate cytokinin production and regulate its concentration and distribution. The *log1/2/3/4/5/7/8* septuple mutant has serious root growth defects (Tokunaga *et al*., 2012). Knocking out *OsCKX4* leads to developmental defects in rice roots (Geng *et al*., 2023). Root-specific expression of *CaCKX6* in chickpea can increase LR development (Khandal *et al*., 2020). Identifying genes that simultaneously regulate vascular tissue and LR development will help to clarify the integration mechanism of these regulatory pathways and provide a new theoretical basis for the breeding of high-yield crops in the future.

AUXIN/INDOLE-3-ACETIC ACID (Aux/IAA) is an auxin early-response gene. Typical Aux/IAA proteins contain four domains. Domain I recruits the co-suppressor TOPLESS (TPL) to inhibit ARF activity (Szemenyei *et al*., 2008). Domain II contains the VGWPP degron motif, which interacts with TRANSPORT INHIBITOR RESPONSE1/AUXIN SIGNALING F-BOX (TIR1/AFB) and enables Aux/IAA protein degradation through the 26S proteasome (Dharmasiri *et al*., 2003; Tan *et al*., 2007). Domains III and IV form heterodimers with ARFs, which inhibit their activity (Korasick *et al*., 2014). Mutations in the VGWPP motif can cause plant growth defects. In Arabidopsis, the *IAA14* (VGWP**S**) mutation causes plant dwarfing, reduced LRs, and geotropism (Fukaki *et al*., 2002). *OsIAA23* (V**E**WPP) mutations cause plant dwarfing, adventitious roots, and root hair reduction in rice (Jun *et al*., 2011). *GmIAA27* (VGW**L**P) mutations cause plant dwarfing and reduced LRs in soybean (Su *et al*., 2022). In maize, *RUM1* encodes the Aux/IAA protein; the *rum1* mutant lacks domain II, resulting in a significant reduction in LRs (Behrens *et al*., 2011). In rapeseed, *BnaA03.IAA7* (VEWP**L**), *BnaC05.IAA7* (VEW**L**P), and *BnaA05.IAA2* (VGWP**S**) mutations led to a dwarf stature (Zhao *et al*., 2019; Huang *et al*., 2020; Ping *et al*., 2022). Mutations in Aux/IAA primarily affect plant height, LR development, and geotropism. To date, it has not been reported that Aux/IAA simultaneously regulates the development of vascular tissue and LR in rapeseed.

Rapeseed (*Brassica napus*) is an important oilseed crop that is distributed worldwide. The development of vascular tissue and LR has a significant effect on the yield of rapeseed. In this study, we identified a novel mutant named *T16* using an ethyl methanesulfonate (EMS) mutant library of rapeseed. *T16* exhibits leaf wilting on sunny days. In addition, it also shows dwarfing and reduced LRs. Dysplasia of the *T16* vascular tissue was observed during histological examination. SEM data indicate that the internode cells were shortened. Map-based cloning and genetic complementarity experiments confirmed that a mutation in the Aux/IAA gene *BnaA03.IAA13* led to the observed phenotypic variations. Transcriptome analysis revealed that auxin and cytokinin signaling pathway-related genes were differentially expressed. Interestingly, although *T16* plants had certain developmental defects, the height of heterozygous plants was significantly decreased without affecting other agronomic traits. Based on the two single nucleotide polymorphisms (SNPs) of *BnaA03.iaa13* in *T16*, we developed cleaved amplified polymorphic sequence (CAPS) markers for breeding. In conclusion, our study reveals an Aux/IAA gene that can simultaneously regulate the development of vascular tissue and LRs, providing novel insights into the genetic basis of the regulation of vascular tissue and LR development in rapeseed. Our results further highlight that the new mutant *T16* has value for practical application as a novel germplasm in semi-dwarf rapeseed breeding.

## Materials and methods

### Plant materials and growth conditions

The mutant *T16* was identified from the EMS mutant library of rapeseed (Tang *et al*., 2020). *T16* was hybridized with ZS11 to construct F_1_, F_2_, and BC_1_ populations for genetic analysis, and with the inbred line B409 (*T16* as recurrent parents) to construct the mapping populations F_2_ and BC_1_–BC_5_. T16 was also hybridized with the inbred line 7112 for yield trait evaluation. The mapping population was planted in Wuhan (29°58ʹN, 113°41ʹE) in October of each year and in Lanzhou (36°03ʹN, 103°40ʹE) in May of each year, with a row spacing of 15 cm and 10 cm and 10–15 plants per row. Yield-related traits were evaluated under field planting conditions. Arabidopsis and tobacco were planted in the greenhouse under conditions of 16 h/8 h light/dark and a constant temperature of 20 °C.

### Paraffin sections

Paraffin sections of plant tissues were used for microscopic observations of vascular tissue. Petioles of plants at five-leaf stage and the stems at the first branch of the initial flowering stage were selected (three biological replicates). Tissues were fixed with 50% formalin–acetic acid–alcohol fixation solution, kept under vacuum for 30 min, and then sectioned according to the method previously described by Li *et al*. (2023). Sections were observed and photographs taken using a microscope (Nikon, Tokyo, Japan). ImageJ was used to determine the number of petiole vascular bundles, number of stem vessels, and radius of the vessels in the stem.

### Scanning electron microscope observations

Stomatal morphology was observed at different times of day. Samples were collected at 08.00 h and 13.00 h, taking the leaves from the third leaf at the five-leaf stage (three biological replicates). A 6 mm diameter punch was used to take two samples from the same part of each leaf. Leaf discs were placed in 2.5% glutaraldehyde for fixation and kept under vacuum for 30 min. Fixed samples were sent to the electron microscope platform of Huazhong Agricultural University for observation (JSM-6390LV). At least three non-overlapping views of each sample were obtained. The total number of stomata in 10 views was counted and all stomatal apertures were measured, and the average stomatal number and aperture for each view was calculated. ImageJ was used to count stomata, measure stomatal apertures and internode cell lengths, and calculate the mean stomatal number and aperture.

### Measurement of photosynthetic parameters

At 08.00 h and 13.00 h, a Li-6800 portable photosynthetic instrument was used to measure the photosynthetic parameters of the third leaf of plants at the five-leaf stage, according to the steps in the operating manual of the instrument. Five biological replicates were performed.

### Map-based cloning

*T16* and the distant inbred line B409 were used to construct a mapping population. The highest- and lowest-stem plants from the F_2_ populations were selected for DNA mix pools (two pools for each phenotype, with 10 plants mixed in each pool); the parents *T16* and B409 were each mixed in one pool. The *Brassica* 50K SNP BeadChip Array and bulked segregant analysis were used for mapping, following the steps described previously (Li *et al*., 2023). WebSat (https://bioinfo.inf.ufg.br/websat/) was used to design simple sequence repeat (SSR) markers, and intron polymorphism markers were designed manually in the intron region. The six DNA mix pools mentioned above were used to screen for polymorphisms of these markers. Then, these polymorphic markers were used to identify recombination events.

### Construction of transgenic plants

The mutated *BnaA03.iaa13* gene sequence was amplified, including the putative promoter region of 1149 bp and the downstream 633 bp. The sequence was cloned into the vector pCMBIA2300 and transformed into normal rapeseed Westar and ZS11 using the hypocotyl impregnation method (Dun *et al*., 2011). Arabidopsis was transformed by the immersion inflorescence method (Clough *et al*., 1998). The SNP1 and SNP2 point mutations were created by site-specific mutagenesis using overlap extension PCR, cloned into the vector pCMBIA2300, and transformed into Arabidopsis.

### Subcellular localization

The coding sequence of *BnaA03.IAA13* and the mutated gene *iaa13* without the stop codon were amplified from the leaf cDNA of ZS11 and *T16*. The fragment was then inserted into the pMDC83 vector in-frame with the green fluorescent protein (GFP) coding sequence, with expression driven by the cauliflower mosaic virus 35S promoter. The resulting construct and marker construct H2B-mCherry were introduced into *Agrobacterium* strain GV3101. The leaves of 3-week-old tobacco plants were infiltrated with a GV3101 cell suspension harboring *IAA13*-GFP and H2B-mCherry. Subcellular localization was observed using a confocal laser microscope (Leica Microsystem, Wetzlar, Germany) 3 d after infiltration.

### Histochemical GUS standing

The putative promoter region (1149 bp) of *BnaA03.IAA13* was cloned into the vector pCMBIA2300-GUS and transformed into Arabidopsis. The positive transgenic plants at the seedling and flowering stages were incubated overnight at 37 °C in X-Gluc solution as a substrate for β-glucuronidase (GUS) and then photographed using a microscope as previously described (Li *et al*., 2019). Multiple independent transgenic lines were observed to ensure the reproducibility of GUS staining results.

### Split-luciferase complementation assay

The coding sequences of *BnaA03.IAA13* and *BnaA03.iaa13* were cloned into the vector JW771-cLUC after deleting the stop codon, and the coding sequence of *TIR1* was cloned into the vector JW772-nLUC after deleting the stop codon. The two vectors were transferred together into tobacco and measured used the NightSHADE EVO LB 985N In Vivo Plant Imaging System (Berthold Technologies, Germany) after 60 h of growth in darkness. Specific steps were as previously described (Liang *et al*., 2022).

### RNA extraction and qRT–PCR

Rapeseed tissues (roots, stems, leaves, buds, siliques, and seeds) and Arabidopsis leaves were collected for RNA extraction (three biological replicates were made). The collected tissues were quickly frozen in liquid nitrogen. Total RNA was extracted using the Total RNA Isolation Kit (TINAGEN, Beijing, China). First-strand cDNA was reversed-transcribed using a RevertAid First Strand cDNA Synthesis Kit (Thermo Fisher Scientific, Waltham, MA, USA). Quantitative real-time (qRT)–PCR reactions were performed on a CFX96 Touch Real-Time PCR detection system (Bio-Rad Laboratories, Hercules, CA, USA) using SYBR Green Supermix (Toyobo, Japan). *BnaActin7* (*BnaC02G0037200ZS*) and *BnaENTH* (*BnaC01G0065000ZS*) were used as internal references in rapeseed; *AtActin2* (*AT3G18780*) and *AtActin7* (*AT5G09810*) were used as internal references in Arabidopsis. Relative expression levels were calculate according to a previously described method (Li *et al*., 2019). Primers used are listed in Supplementary Table S1.

### Transcriptome analysis

The roots and stems from Arabidopsis transgenic lines (T_3_ generation) were used for RNA sequencing (RNA-seq) with three biological replicates. The collected tissues were quickly frozen in liquid nitrogen. The RNA used in RNA-seq was applied to test the transcriptome data by qRT–PCR. Total RNA was isolated using TRIzol Reagent (Invitrogen), and transcriptomes were performed on an Illumina NovaSeq 6000 platform by Shanghai Personal Biotechnology Cp (Shanghai, China). The sequencing data were filtered using the fastp software by removing low-quality reads and adapters (Chen *et al*., 2018). Clean reads were mapped to the Arabidopsis genome (https://www.arabidopsis.org) using TopHat2 (Kim *et al*., 2019). Fragments per kilobase million reads values were used to calculate gene expression levels using featureCounts (Liao *et al*., 2014). Differentially expressed genes (DEGs) were determined using DESeq2 with thresholds of log_2_ (fold change) >1 and a significant *P* value <0.05 (http://www.bioconductor.org/packages/release/bioc/html/DESeq2.html). Functional annotation, classification, and pathway enrichment analyses were carried out using the Gene Ontology (GO) (http://www.geneontology.org) and Kyoto Encyclopedia of Genes and Genomes (KEGG) (https://www.kegg.jp/) databases.

### Determination of cytokinin concentration

After vacuum freeze-drying, samples of roots and stems from *T16* and ZS11 at the cotyledon stage were ground into powder. Samples of 100 mg of the powder were accurately weighed and dissolved in 1 ml of 70% methanol, The mixture was vortexed once every 30 min for 30 s, repeated six times, and then placed at 4 °C overnight. Then, the samples were centrifuge at 4 °C at 13 900 *g* for 10 min; the supernatant was filtered (0.22 μm pore size filter) and then analyzed using LC-MS. The standard samples for detecting *N*^6^-(Δ2-isopentenyl)-adenine and *trans*-zeatin were used to draw standard curves (0.03125, 0.0625, 0.0125, 0.25, 0.5, 1, 2, 4, 8, 16, 32, 64, 128 ng ml^–1^). Three independent experiments were performed for each assessment.

### Phylogenetic analysis and gene evolution analysis

The Aux/IAA protein sequences of rapeseed and Arabidopsis were downloaded from the Brassicaceae Database (http://www.brassicadb.cn). Phylogenetic analysis was carried out with MEGA7; the neighbor-joining tree was constructed with bootstrap values tested for 1000 trails; MEME was used to find the conserved motif of Aux/IAA proteins. TBtools was used to visualize the phylogenetic tree and motif analysis. Genomes of algae, bryophytes, and pteridophytes were downloaded from NCBI, and the Aux/IAA protein conservative domain was used as the search object for a genome-wide search. MEGA7 and GENEDOC were used for protein sequence analysis.

### Statistical analysis

Quantification analyses on all the measurements were conducted in GraphPad Prism 8. Statistically significant differences were identified using Student’s *t*-test.

## Results

### *T16* exhibits lateral root development defects, leaf thermal sensitivity, and dwarfism

**Fig. 1.**
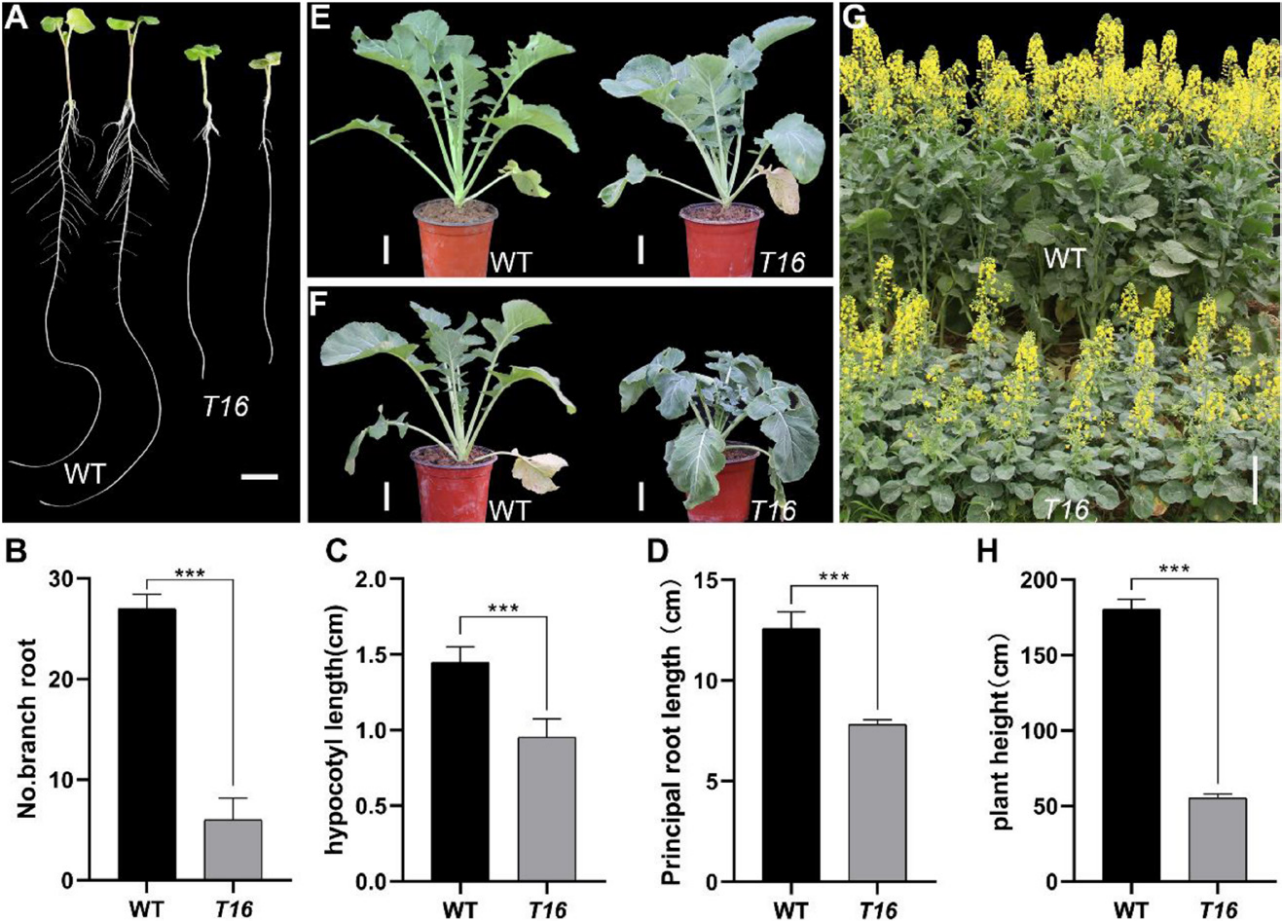
Phenotypic characterization of *T16*. (A) Comparison of the hypocotyl and root of *T16* and the wild type (WT) ZS11. Bar=1 cm. (B–D) Statistical comparisons of the number of lateral roots (B), hypocotyl length (C), and principal root length (D) in *T16* and WT plants. (E, F) Leaf phenotype at 09.00 h (E) and 13.00 h (F), showing wilting of *T16.* Bars=5 cm. (G) Comparison of plant height during the flowering period. Bar=25 cm. (H) Comparison of plant height at maturity. Values in (B–D, H) are means ±SD (*n*≥15). Asterisks indicate statistically significant differences (***P<0.001; Student’s *t-*test).

The *T16* mutant was screened using the EMS mutation library of ZS11 [a Chinese open-pollination cultivar, wild type (WT)]. Compared with the WT, the hypocotyl of *T16* was decreased in length by 36.2%, the main root was shortened by 37.9%, and the number of LRs was decreased by 77.8% (*P<*0.001) (Fig. 1A–D). *T16* also exhibited leaf wilting on sunny days. Before 09.00 h, there was no difference in leaf phenotype between *T16* and the WT (Fig. 1E). As the temperature increased, *T16* leaves gradually wilted, and the most notable wilting phenotype was observed at ~13.00 h (Fig. 1F). In the evening, the leaf phenotype of *T16* recovered so that it showed no difference from that of the WT. When grown in a greenhouse (22 °C), the phenotype of *T16* leaves did not differ from that of the WT. When the plants were transferred outdoors (32 °C), *T16* leaves wilted very quickly (within ~5 min), whereas the WT leaves did not show significant changes (Supplementary Video S1). After transferring the plants back to the greenhouse, the wilted leaves of *T16* gradually returned to normal (Supplementary Video S2). In addition, *T16* showed dwarfism throughout the whole growth period. At maturity, the plant height of *T16* was only 55 ± 3.5 cm, which is ~30% of the height of the WT (181 ± 6.4 cm) (Fig. 1G, H). In summary, the phenotypic variation in *T16* exhibits pleiotropy, affecting root development, hypocotyl elongation, leaf morphology, and plant height.

### Abnormal development of vascular tissue and stem in *T16*

**Fig. 2.**
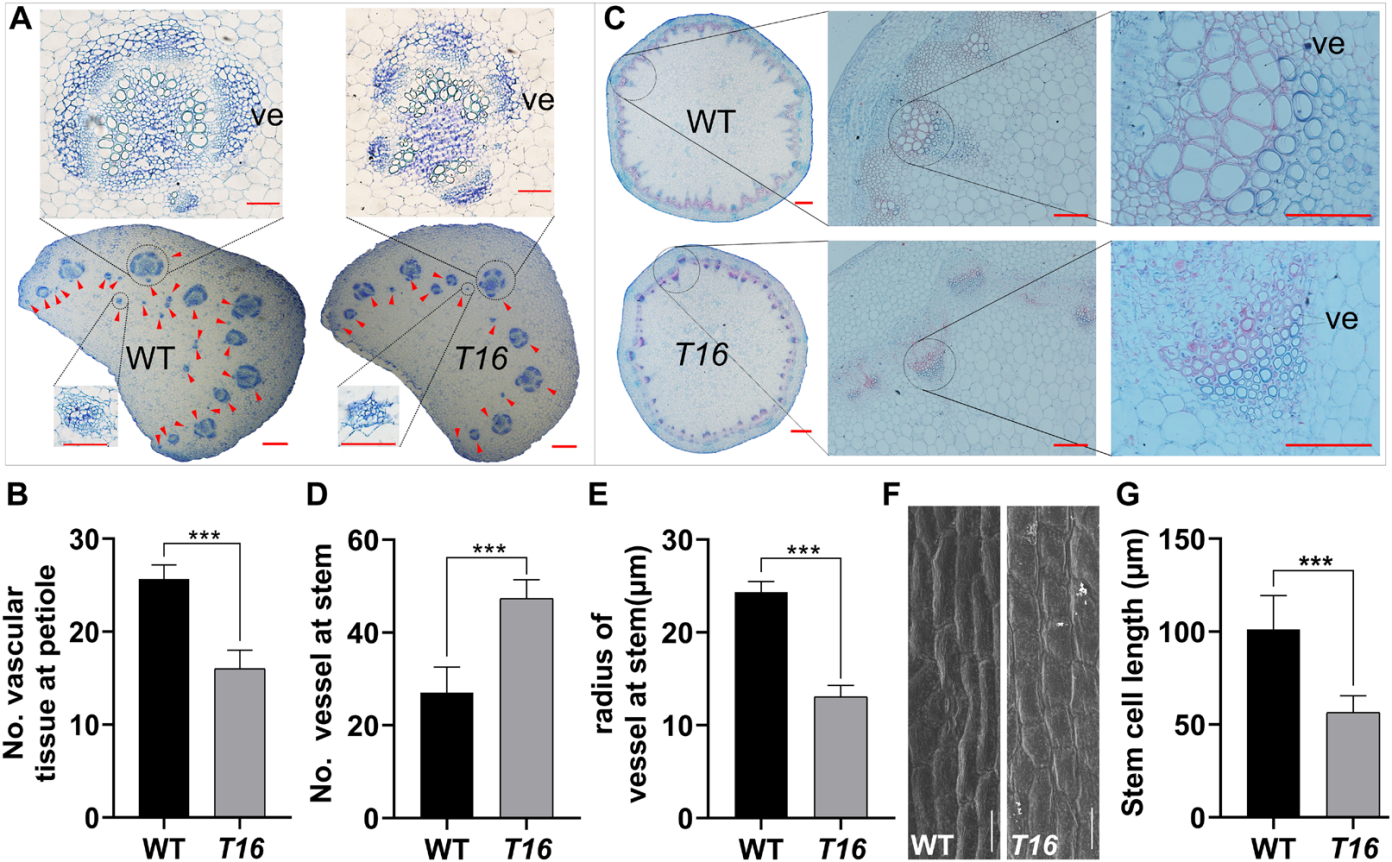
Analysis of histological sections of wild-type (WT) and *T16* rapeseed plants. (A) Microscopic observation of paraffin sections of petiole vascular bundles. The red arrowheads indicate individual vascular bundles. ve, vessel. Bars=1 mm in the main images; 500 μm in the insets. (B) Statistical comparison of the number of vascular bundles in petioles of the WT and *T16*. (C) Microscopic observation of stem cross sections. Bar = 1 mm in the main images; 100 μm in the insets. (D, E) Statistical comparison of the number of stem vessels (D) and the radius of the vessels in the stem (E) of the WT and *T16*. (F) SEM observation of internode cell size. Bars=50 mm. (G) Statistical comparison of internode cell length in the WT and *T16*. Three biological replicates were performed for the cytological observations. Values in (B, D, E, G) are means ±SD (*n*=15). Asterisks indicate statistically significant differences (***P<0.001; Student’s *t-*test).

To further understand the *in vivo* variation in *T16*, we observed leaf stomatal morphology in the morning (09.00 h) and middle of the day (13.00 h) using SEM. Electron microscopic observations showed that the stomatal density did not differ between *T16* and the WT, and there was no significant difference in stomatal aperture between 09.00 h and 13.00 h (Supplementary Fig. S1A–J). Further measurements of photosynthetic parameters indicated no significant differences between *T16* and the WT in their transpiration rate and stomatal conductance (Supplementary Fig. S1K, L), indicating that the dynamic changes in *T16* leaf wilting were unrelated to stomatal development and movement. Microscopic observation of paraffin-embedded sections of petioles showed that the number of vascular bundles in the *T16* petiole was significantly lower than that in the WT (Fig. 2A, B). The number of xylem vessels in the vascular bundle was significantly higher, and the vessel radius was significantly smaller, in *T16* (*P<*0.01) (Supplementary Fig. S2). The stem from the first internode of the initial flowering stage was selected for paraffin sectioning. When observed under a stereomicroscope, large vessels could be observed in the WT but not in *T16*, and the transverse radius of the cut WT stem was significantly larger than that of *T16* (Fig. 2C). Examination under the optical microscope showed that the number of vessels was significantly greater in *T16* than in the WT, but the vessel radius of *T16* was significantly smaller. The vessel radius of the WT was larger than the radius of the surrounding tracheid, but the radius of most vessels in *T16* did not differ from that of the surrounding tracheid (Fig. 2C–E). These results indicate that the development of vascular tissue is defective in *T16* and the development of the vessel radius is insufficient. Poiseuille’s law states that the hydraulic conductivity of a conduit is proportional to the fourth power of the vessel radius. Therefore, the leaf wilting of *T16* is due to insufficient water supply caused by the inadequate development of the xylem vessel radius. In addition, SEM observations of internode cells in mature plants showed that the cell length of *T16* was 44.3% shorter (*P<*0.001) compared with that of the WT (Fig. 2F, G), indicating that the dwarfism of *T16* is caused by severe inhibition of cell elongation.

### The phenotype of *T16* is controlled by a single nuclear gene located on chromosome A03

**Fig. 3.**
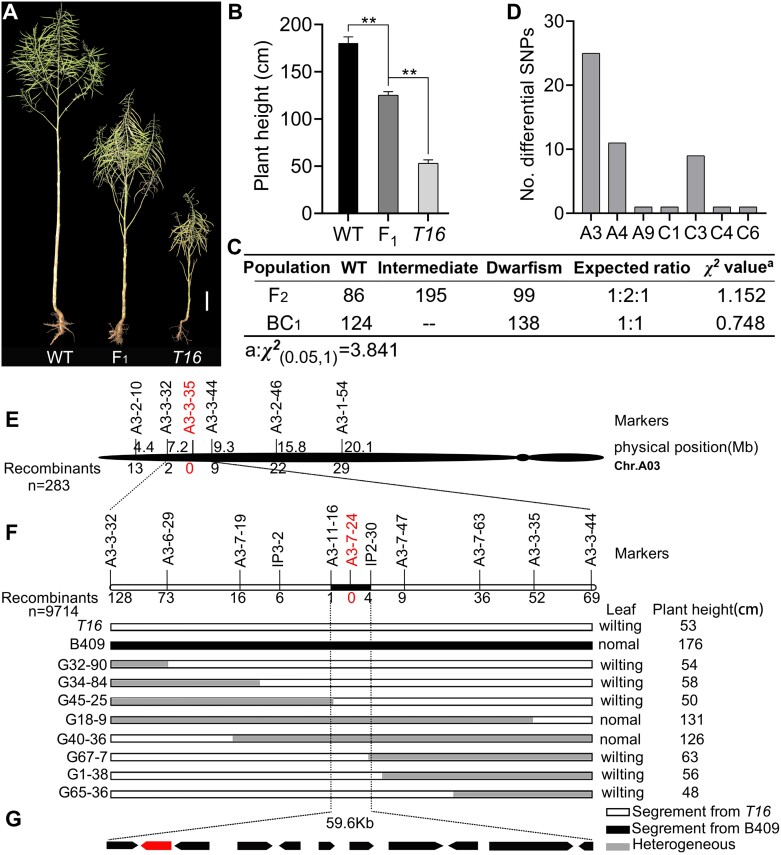
Genetic analysis and map-based cloning. (A) Phenotypes of wild type (WT), F_1_, and *T16* plants at maturity. Bar=10 cm. (B) Statistical analysis of plant height at maturity (*n*=15). Asterisks indicate statistically significant differences (**P<0.01; Student’s *t-*test). (C) Plant height segregation ratio of the F_2_ and BC_1_ populations at maturity. (D) Chromosomal position information of differential SNPs obtained by using *Brassica* 50 K SNP BeadChip Array analysis. (E) Preliminary mapping of the functional gene. Identified candidate segments occupy a region of 2.1 Mb on ChrA03. (F) Fine mapping of the functional gene. Identified candidate segments occupy a region of 59.6 kb. (G) The 59.6 kb candidate region contains 11 genes, with *BnaA03.IAA13* indicated in red.

To ensure the accuracy of phenotype identification, the plant height at maturity was used for the analysis of genetic relationships. A significant difference in plant height was observed between the hybrid F_1_ and its parents (*P<*0.01), but no difference was detected compared with the mid-parent value (Fig. 3A, B). Analysis of plant height at maturity showed that the segregation ratio of F_2_ and BC_1_ populations was 1:2:1 and 1:1, respectively (Fig. 3C), indicating that the phenotype of *T16* is controlled by a semi-dominant nuclear gene.

Using bulked segregation analysis and the *Brassica* 50K SNP BeadChip Array, a total of 49 significantly associated SNPs were identified, mainly enriched on chromosome A03 (ChrA03), ChrA04, and ChrC03 (Fig. 3D; Supplementary Table S2). Then, SSR markers were designed in the regions covered by the ChrA03, ChrA04, and ChrC03 polymorphic SNPs, using the six DNA mix pools to identify whether these SSR markers have polymorphisms. The results showed that polymorphic markers were successfully identified only in the ChrA03 regions. These polymorphic markers were used to identify the recombination events in 283 dwarf plants from the F_2_ and BC_1_ populations. The results showed that ~2.1 Mb between markers A3-3-32 and A3-3-44 were candidate regions, and a co-segregation marker, A3-3-35, was screened (Fig. 3E).

### Fine mapping and candidate gene analysis

To improve the efficiency and accuracy of fine mapping, only plants with wilted leaves were used to identify recombinant events at the vegetative stage. We examined the plant height of these potential recombinant plants at maturity. Only dwarf plants were determined to be true recombinant plants. Based on this standard, recombination identification was performed on 9714 dwarf plants, comprising 1504 BC_2_F_1_, 2410 BC_3_F_1_, 2800 BC_4_F_1_, and 3000 BC_5_F_1_ individuals. The final candidate region was determined to be a region of ~59.6 kb between markers A3-11-16 (one recombinant plant) and IP2-30 (four recombinant plants), while co-isolating at marker A3-7-24 (Fig. 3F). As expected, the self-pollinated progenies of the last five recombinant plants showed the same dwarfism and leaf wilting phenotypes as those of *T16* (Supplementary Fig. S3), supporting the reliability of the fine-mapping results.

**Fig. 4.**
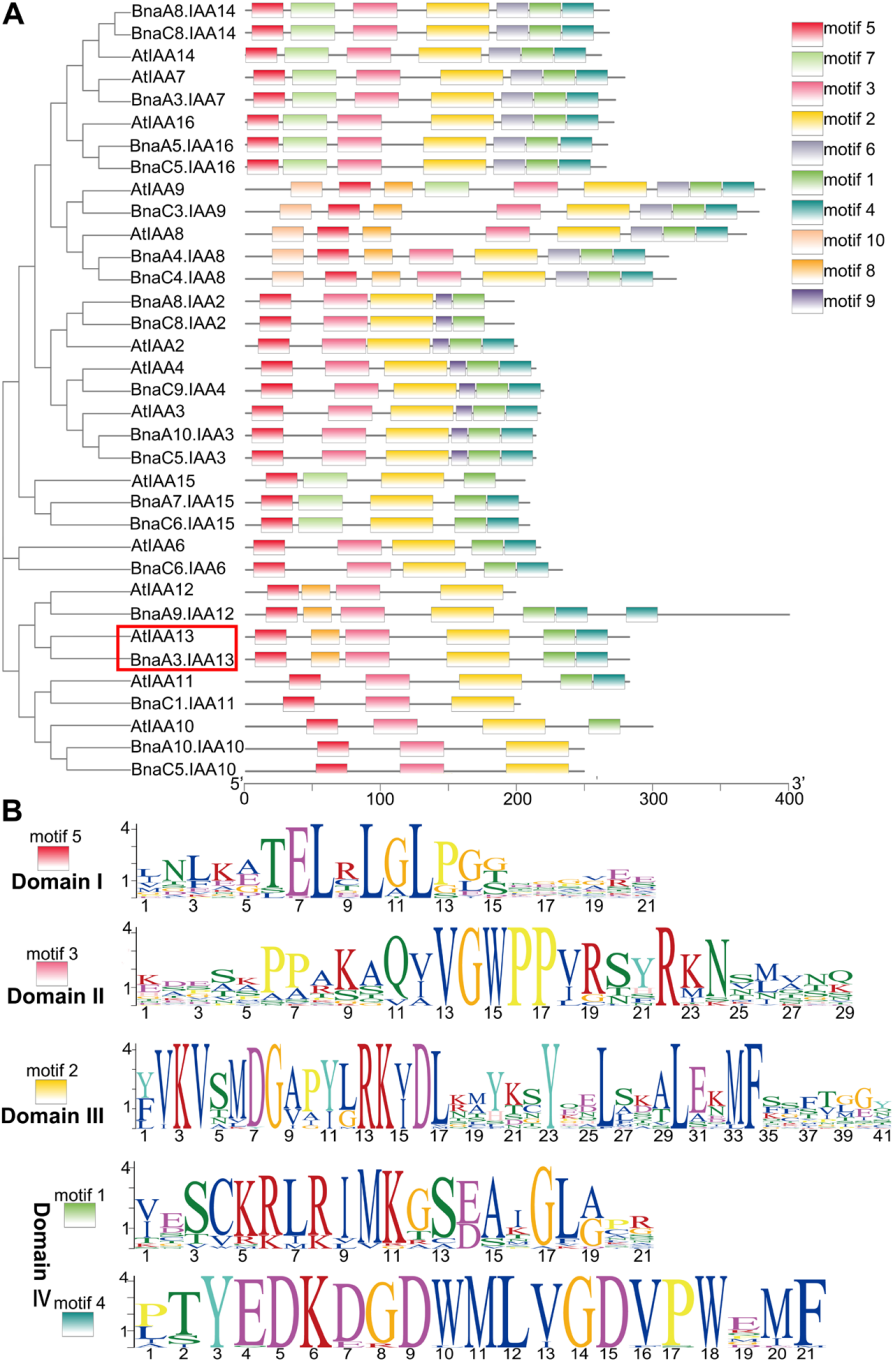
Phylogenetic analysis of Aux/IAA proteins. (A) Phylogenetic tree of 14 IAA genes in Arabidopsis and 21 IAA genes in rapeseed, also showing the distribution of 10 motifs on Aux/IAA proteins. Analysis showed that *BnaA03.IAA13* in rapeseed is homologous to *IAA13* in Arabidopsis (highlighted with the red box). (B) Five motifs representing four conserved domains were distributed on the Aux/IAA proteins.

**Fig. 5.**
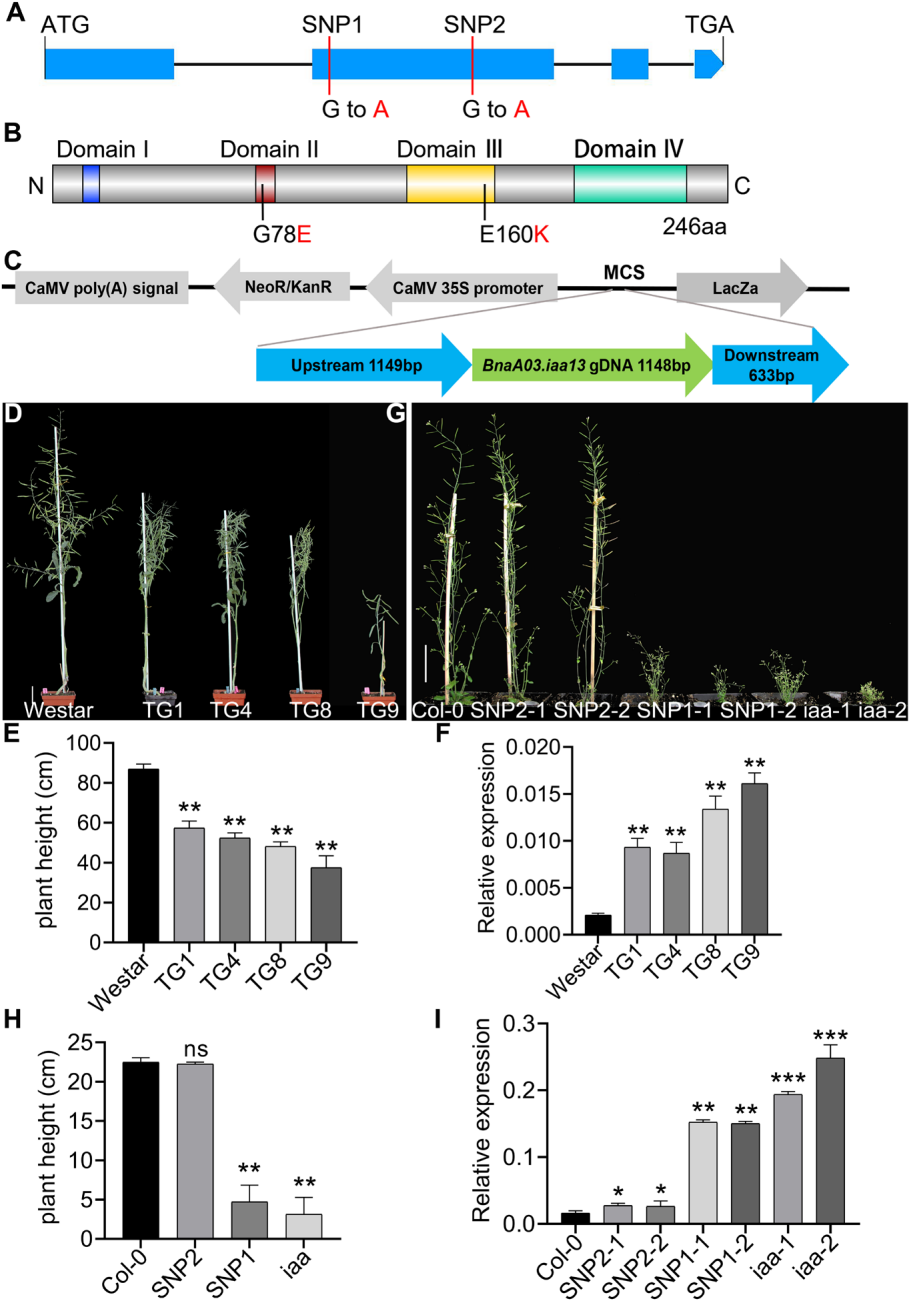
Verification of genetic complementarity. (A) The gene structure of *BnaA03.IAA13* showing the locations of the SNPs. (B) The protein structure of BnaA03.IAA13 showing the locations of mutated amino acids resulting from the SNPs. (C) Complementary vector structure diagram. The vector was constructed using pCMBIA2300 as the vector backbone, inserting the mutated gene *BnaA03.iaa13*, including its upstream promoter region of 1149 bp and downstream 633 bp. The complementary vectors of SNP1 and SNP2 were created through targeted mutagenesis. (D) Phenotype of *BnaA3.iaa13* rapeseed transgenic plants at maturity. A total of 26 transgenic lines were obtained, and phenotypic analysis was performed on four representative lines. Bar=5 cm. (E) Statistical analysis of plant height in rapeseed transgenic lines (*n*=10). (F) qRT–PCR analysis of *BnaA03.iaa13* expression levels in rapeseed transgenic lines (*n*=3). *BnaActin7* was used as a reference gene. (G) Phenotype of *BnaA3.iaa13* Arabidopsis transgenic plants. At least 10 independent transgenic lines were obtained for each vector, and the transgenic plants of each vector had similar phenotypes. Bar=5 cm. (H) Statistical analysis of plant height in Arabidopsis transgenic lines (*n*=10). (I) qRT–PCR analysis of *BnaA03.iaa13* expression levels in Arabidopsis transgenic lines (*n*=3). *AtActin2* was used as a reference gene. The values in the bar charts represent the mean ±SD. Asterisks indicate statistically significant differences (*P<0.05, **P<0.01, ***P<0.001; Student’s *t-*test).

There were 11 annotated genes in the 59.6 kb candidate fragment of the ZS11 genome (Fig. 3G). A comparison of the DNA sequences of these 11 candidate genes in the WT and *T16* showed that eight genes exhibited no variation, whereas three genes did (Supplementary Table S3). *BnaA03T0160700ZS* and *BnaA03T0160900ZS* had a point mutation in the simple repeat sequence of the intron, which did not lead to a change in the predicted protein sequence. *BnaA03T0160600ZS* had two single-nucleotide substitutions (G-to-A) in its second exon. *BnaA03T0160600ZS* contains four exons and three introns, encoding the Aux/IAA protein BnaA03.IAA13, comprising 246 amino acids. Phylogenetic analysis showed that BnaA03.IAA13 is homologous to Arabidopsis IAA13 and contains four complete conserved domains (Fig. 4), indicating that BnaA03.IAA13 is a typical Aux/IAA protein. The natural population contained 32 SNP haplotypes in *BnaA03.IAA13* and the two SNP variations of *T16* were unique. The two SNPs led to changes in the amino acid sequence of BnaA03.IAA13 (Supplementary Fig. S4). SNP1 causes an amino acid substitution of glycine with glutamic acid at position 79 (designated as G79E), and SNP2 causes substitution of glutamic acid with lysine at position 160 (designated as E160K) (Fig. 5A, B). The G79E mutation is located in the degron motif VGWPP; mutations in this motif can lead to an auxin-deficient phenotype such as dwarfism (Fukaki *et al*., 2002; Jun *et al*., 2011). Based on these results, *BnaA03.IAA13* was determined to be the candidate gene.

### *BnaA03.IAA13* is the functional gene, and G79E is the functional site

To verify whether the variation in *BnaA03.IAA13* led to the semi-dominant trait of *T16*, a complementary vector for the mutated gene *BnaA03.iaa13* was constructed (Fig. 5C). A significant decrease in plant height and mild leaf wilting was observed in the T_0_ generation positive plants transformed into the normal material Westar and ZS11, and validated in the T_1_ generation plants (Fig. 5D, E; Supplementary Fig. S5). In addition, the phenotypic variation in transgenic plants was positively correlated with the abundance of *BnaA03.iaa13* expression revealed by qRT–PCR analysis (Fig. 5F; Supplementary Fig. S6A). Similar phenotypes were observed in Arabidopsis transgenic plants (Fig. 5G–I; Supplementary Fig. S6B). These results confirmed that the mutation of *BnaA03.IAA13* causes a phenotypic variation in *T16* and is positively related to the abundance of *BnaA03.iaa13* expression.

To verify which SNP was the functional site, complementary vectors for SNP1 (G79E) and SNP2 (E160K) were constructed by site-directed mutagenesis and introduced into Arabidopsis. The results showed that the phenotype of the SNP1 transgenic plants was similar to that of the *BnaA03.iaa13* transgenic plants, both of which showed small and curly leaves, short roots, few LRs, severe dwarfing, and loss of apical dominance. However, the phenotype of the SNP2 transgenic plants was not different from that of Col-0 (Fig. 5G). In addition, the sequence alignment of the *IAA13* homologous gene in several species showed that SNP1 is located at the degron motif, which was highly conservative. SNP2 is located at the last lysine of the α2 motif. This site contains multiple amino acid types, indicating that it was not conserved during evolution (Supplementary Fig. S7). These results suggest that G79E is the functional site, and E160K is not.

### *BnaA03.IAA13* is highly expressed in the root and stem, and its protein is localized in the nucleus

**Fig. 6.**
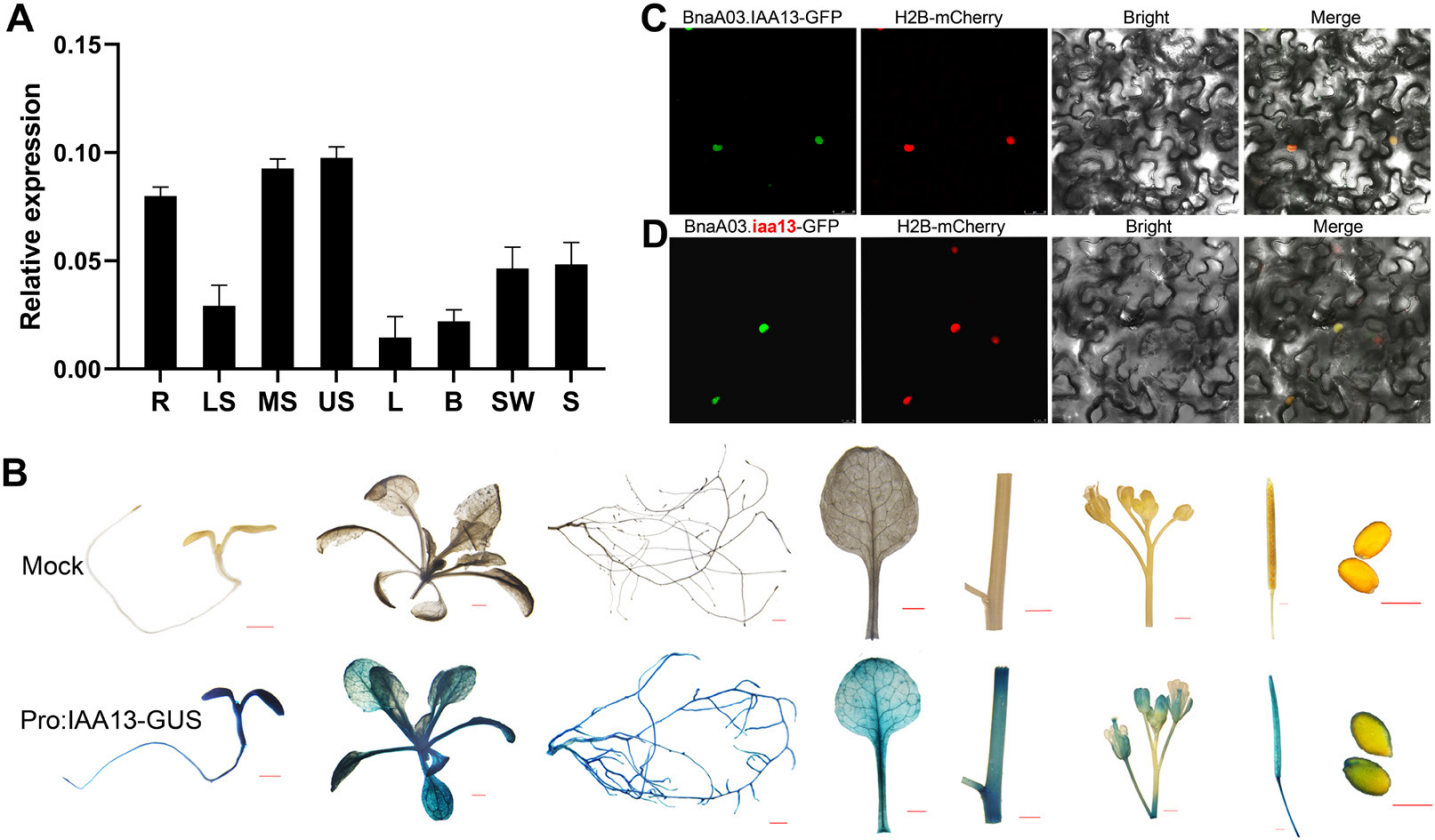
Expression pattern analysis of *BnaA03.IAA13*. (A) Expression analysis of *BnaA03.IAA13* in various tissues of rapeseed, using *BnaActin7* as a reference gene, with three biological replicates. R, root; LS, lower stem; MS, middle stem; US, upper stem; L, leaf; B, bud; SW, silique wall; S, seed. (B) GUS staining of different tissues in transgenic Arabidopsis plants. Expression of the *GUS* reporter gene was driven with the *BnaA03.IAA13* promoter; four independent transgenic lines were observed and similar GUS staining results were obtained. Bar=1 mm. (C, D) Subcellular localization of BnaA3.IAA13 (C) and mutated protein BnaA3.iaa13 (D) in tobacco. H2B-mCherry was used as a nucleus-localized marker.

qRT–PCR analysis showed that *BnaA03.IAA13* was expressed in different tissues, with high levels of expression in the roots and stems (Fig. 6A; Supplementary Fig. S8). The expression of the *GUS* reporter gene was driven by the *BnaA03.IAA13* promoter and introduced into Arabidopsis. GUS staining results were similar to the qRT–PCR analysis results: strong GUS signals were detected in roots, hypocotyls, cotyledons, and young rosette leaves, and weak GUS signals were identified in sepals, stigmas, siliques, and episperms (Fig. 6B). To better understand the mechanism of action of *BnaA03.IAA13*, the coding sequence was fused with the coding sequence of GFP and transiently transformed into tobacco. The results showed that *BnaA03.IAA13*-GFP co-localized with the nuclear localization signal H2B-mCherry (Fig. 6C), indicating that BnaA03.IAA13 is a nuclear localization protein that may participate in the TIR1/AFB complex-mediated IAA signaling pathway. In addition, the mutated protein BnaA03.iaa13 was also located in the nucleus, indicating that the amino acid change did not affect protein localization (Fig. 6D).

### Mutations in *BnaA03.IAA13* affect gene expression related to multiple signaling pathways

**Fig. 7.**
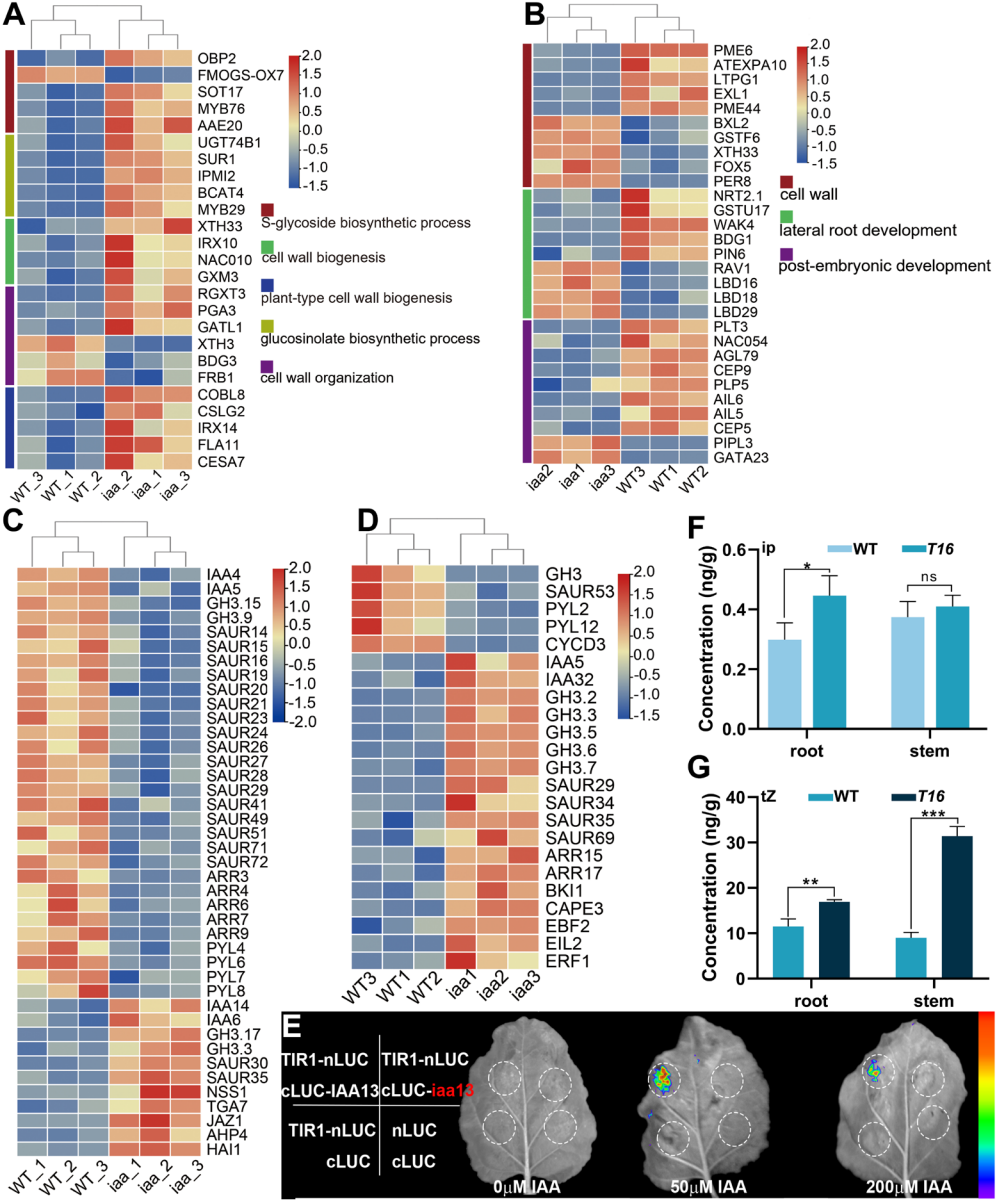
Transcriptome analysis of the stem and root of Arabidopsis transgenic plants, and interaction validation and cytokinin content analyses. (A) Gene expression heatmap of genes expressed in the stem enriched in GO terms related to the cell wall and glucosinolate biosynthetic process. (B) Gene expression heatmap of genes expressed in the root enriched in GO terms related to the cell wall and root development. (C) Gene expression heatmap of genes expressed in the stem enriched in KEGG pathways related to plant hormone signal transduction. (D) Gene expression heatmap of genes expressed in the root enriched in KEGG pathways related to plant hormone signal transduction. (E) A luciferase complementation assay confirmed the interaction between TIR1 and BnaA03.IAA13. (F, G) *N*^6^-(Δ2-isopentenyl)-adenine (ip) and *trans*-zeatin (tZ) concentrations in the root and stem. Values represent the mean ±SD (*n*=4). Asterisks indicate statistically significant differences (*P<0.05, **P<0.01, ***P<0.001; Student’s *t-*test).

To reveal the molecular basis of *BnaA03.IAA13* involvement in vascular tissue and LR development, transcriptome analysis was conducted on the stems and roots of transgenic Arabidopsis lines. In the stems, 994 genes were up-regulated and 300 genes were down-regulated (Supplementary Fig. S9A); in the roots, 600 genes were up-regulated and 551 genes were down-regulated (Supplementary Fig. S9B). The reliability of the transcriptome data was confirmed using qRT–PCR validation of DEGs (Supplementary Fig. S9C–F). In the stems, GO enrichment showed that DEGs were enriched in biological processes such as ‘cell wall organization’, ‘cell wall biogenesis’, and ‘plant-type cell wall biogenesis’ (Fig. 7A; Supplementary Fig. S10A), indicating that *BnaA03.IAA13* may regulate the development of vascular tissue by regulating cell wall biogenesis. In the roots, GO showed enrichment in ‘cell wall’, ‘lateral root development’, and ‘post-embryonic root development’ (Fig. 7B; Supplementary Fig. S10B; Supplementary Table S6), indicating that *BnaA03.IAA13* affects the biological processes of LR formation. Interestingly, DEGs were significantly enriched in pathways such as ‘*S*-glycoside biosynthetic process’, ‘glucosinolate biosynthetic process’ and amino acid synthesis (Fig. 7A; Supplementary Fig. S10A, C). The results of previous studies showed that auxin-related factors *IAA13*, *IAA28*, and *ARF2* are coordinators of sulfur metabolism reactions (Falkenberg *et al*., 2008). Our results support this view, indicating that underdeveloped LRs and vascular tissues result in the abnormal absorption and transport of nutrients.

The KEGG enrichment showed that DEGs in both the stem and root were enriched in ‘Plant hormone signal transformation’ (Supplementary Fig. S10C, D), of which the vast majority were auxin early-responsive genes (Aux/IAA, GH3, SAUR) (Fig. 7C, D; Supplementary Tables S4, S5). BnaA03.IAA13 is a typical Aux/IAA protein that contains four complete domains (Fig. 5B). The luciferase complementation assay confirmed that it can interact with TIR1 (Fig. 7E). These results indicate that *BnaA03.IAA13* participates in the classic auxin signaling pathway and may regulate downstream signaling through the IAA13–ARF module. In addition, genes related to the cytokinin signaling pathway were also differentially expressed (Fig. 7C, D; Supplementary Tables S4, S5), indicating that *BnaA03.IAA13* may integrate auxin and cytokinin signaling pathways. CKXs irreversibly degrade cytokinin to maintain cytokinin homeostasis (Kowalska *et al*., 2010); *CKX5* is down-regulated in the stem, whereas *CKX1* and *CKX7* are down-regulated in the root (Supplementary Fig. S9C–F). Multiple AUXIN RESPONSE ELEMENTS (AuxREs) exist in the CKX promoter region, and expression pattern analysis showed that CKXs were expressed in all tissues (Supplementary Fig. S11). Hormone analysis showed a significant increase in the *N*^6^-(Δ2-isopentenyl)-adenine and *trans*-zeatin concentrations in the mutant (Fig. 7F, G). These results suggest that CKXs may be directly regulated by the IAA13–ARF module, thereby affecting cytokinin homeostasis. In addition, multiple genes involved in LR development, such as genes encoding LBDs, SHI-RELATED SEQUENCEs (SRSs), and WOXs, were differentially expressed in the mutants (Supplementary Table S7). The ARF–LBD module regulates LR formation (Okushima *et al*., 2007; Goh *et al*., 2012; Porco *et al*., 2016). The *SRS* gene family regulates plant growth by influencing hormone signaling pathways. *SRS1/LRP1* plays a negative regulatory role in LR development by regulating auxin synthesis (Smith *et al*., 1995; Singh *et al*., 2020). *SRS5* directly binds to the *LBD16/19* promoter to regulate LR development and is directly regulated by *ARF7/19* (Yuan *et al*., 2020). The WOX–ARF module initiates different root types (Brackmann *et al*., 2018; Smetana *et al*., 2019; Zhang *et al*., 2023). In summary, these results indicate that *BnaA03.IAA13* directly or indirectly regulates the expression of genes related to vascular tissue and LR development.

### *BnaA03.iaa13* heterozygotes show reduced plant height without impact on yield

**Fig. 8.**
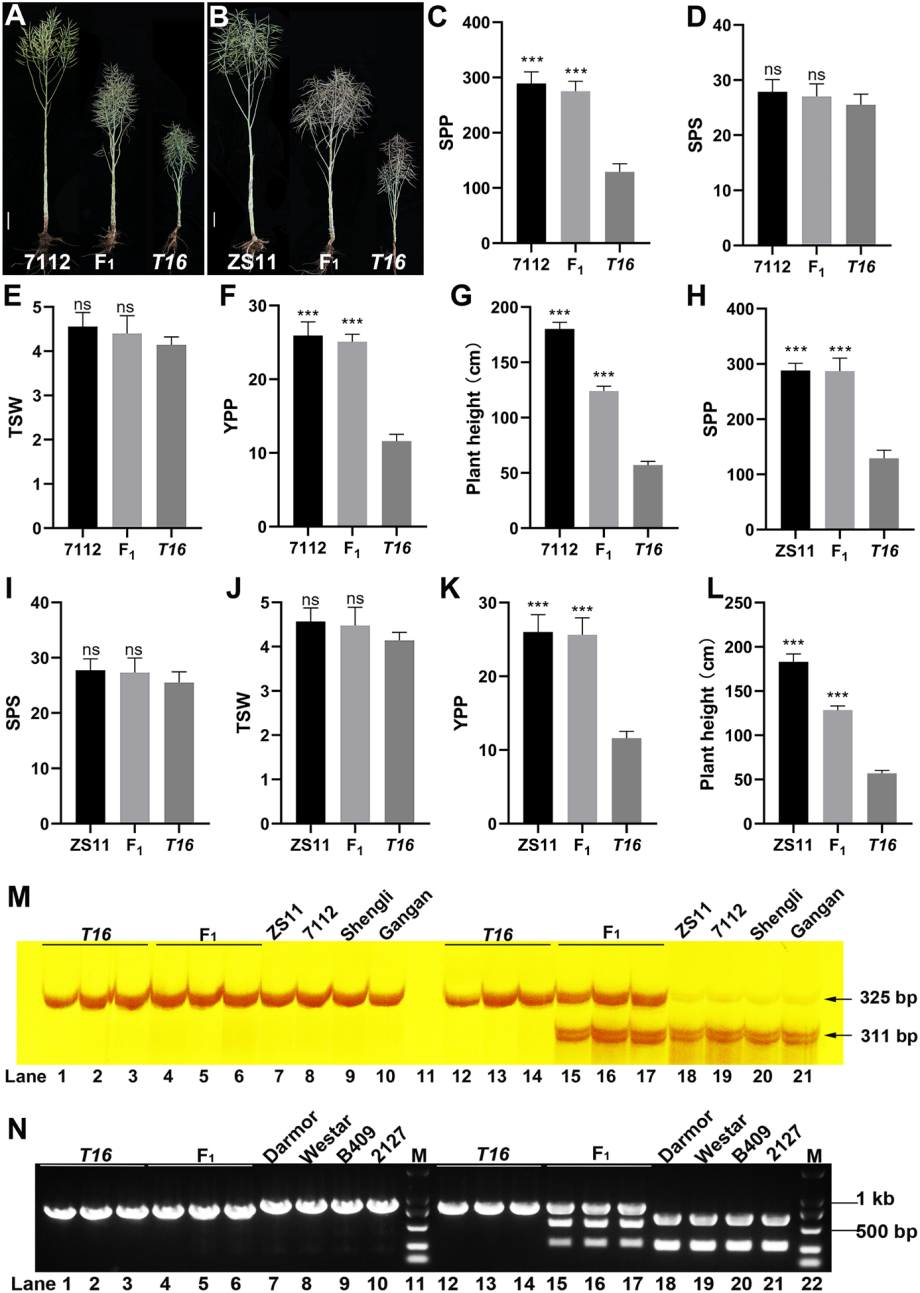
Testing and verification of the value of *T16* for practical breeding. (A) The phenotype of F_1_ plants produced by hybridization between *T16* and the elite inbred line 7112 at maturity. (B) The phenotype of F_1_ plants produced by hybridization between *T16* and the elite variety ZS11 at maturity. (C–G) Investigation of the yield of hybrid offspring produced by crosses between *T16* and 7112. SPP, siliques per plant; SPS: seeds per silique; TSW: thousand-seed weight; YPP: yield per plant. (H–L) Investigation of the yield of hybrid offspring produced by crosses between *T16* and ZS11. Values in (C–L) are means ±SD (*n* =15). Asterisks indicate statistically significant differences (***P<0.001; Student’s *t-*test). (M) A derived cleaved amplified polymorphic sequence marker developed based on SNP1. Polyacrylamide gel electrophoresis detection after digestion with the restriction endonuclease *Bsa*BI. Lanes 1–10 are before digestion and lanes 12–21 are after digestion; lanes 7–10 are different varieties. (N) A CAPS marker developed based on SNP2; agarose gel electrophoresis detection after digestion with the restriction endonuclease *Brs*I digestion.

The F_1_ hybrid derived from *T16* had a plant height suitable for rapeseed production (Fig. 3A), indicating that the semi-dominant characteristic of *BnaA03.iaa13* has practical value for breeding. To further reveal the value of *T16* in breeding, it was hybridized with the high-stem variety ZS11 and inbred line 7112 (with different genetic backgrounds, with plant height in both exceeding 180 cm). Observation of 8-day-old seedlings showed a decrease in the number of LRs in the hybrid, but a serious reduction, similar to that in *T16*, was not observed (Supplementary Fig. S12). In addition, the height of hybrid plants (~130 cm) was significantly shorter than that of the WT (*P<*0.001) (Fig. 8A, B); this height is very suitable for the mechanized production of rapeseed (Fu *et al*., 2013). Most importantly, the yields of the hybrid and elite cultivars did not differ (Fig. 8C–L).

Two SNP variations in *BnaA03.iaa13* were unique to *T16* (Supplementary Fig. S4A). Therefore, two CAPS markers were developed based on these two SNPs. First, primers (CAPS-IAA13) were designed to specifically amplify the two SNPs, with an amplification fragment of 829 bp. For SNP1, a new reverse primer, dCAPS-SNP1-R, was designed at this site and combined with the forward primer CAPS-IAA13-F to amplify a 325 bp fragment from the above-mentioned 829 bp fragment. The restriction endonuclease *Bsa*BI (digestion site GATNNNNATC) was used to digest the 325 bp fragment. Digestion resulted in the production of 311 bp and 14 bp fragments. Detection by polyacrylamide gel electrophoresis showed that the respective sequence of *T16* could not be digested. Different normal plant materials (ZS11, 7112, Shengli, and Gangan) still contained a 311 bp fragment after digestion, whereas the F_1_ hybrid contained 325 bp and 311 bp fragments. After digestion, the 14 bp fragment was too small to be displayed (Fig. 8M). For SNP2, this site was the restriction endonuclease *Bsr*I recognition site (digestion site ACTGG). The use of *Bsr*I to digest the 829 bp fragment produced 549 bp and 280 bp fragments. The results of agarose gel electrophoresis showed that the respective sequence of *T16* could not be digested. Different normal plant materials (Darmor, Westar, B409, and 2127) contained two fragments whereas the hybrid contained three fragments (Fig. 8N). In summary, these two CAPS markers can be used to distinguish between different alleles of *BnaA03.iaa13* and could be used for subsequent molecular breeding.

## Discussion

Understanding the molecular mechanisms underlying vascular tissue and LR development is important to ensure crop safety. Evidence shows that complex molecular mechanisms regulating vascular tissue development involve small peptides, transcription factors, and hormone signaling (Etchells *et al*., 2010; Smetana *et al*., 2019; Fujiwara *et al*., 2023). LRs originate from the central sheath of other roots (Casimiro *et al*., 2003; Singh *et al*., 2023), and their molecular mechanism has been extensively studied and is significantly inflected by hormonal signals (Tokunaga *et al*., 2012; Kang *et al*., 2013; Cavallari *et al*., 2021; Lv *et al*., 2021). Although there have been many reports on the development of vascular tissue and LRs, there is a lack of published data on the genes that simultaneously affect vascular and LR development. On the one hand, it is difficult to obtain this type of mutant material; on the other hand, the development of vascular tissue and LRs is very vulnerable to environmental impacts, resulting in difficult phenotypic identification and a heavy workload. In this study, the *T16* mutant exhibited leaf wilting on sunny days; it also showed dwarfing and reduced LRs (Fig. 1). A developmental defect of vascular tissue was the cause of the leaf wilting (Fig. 2). Map-based cloning and transgenic complementary verification confirmed that the mutation in *BnaA03.IAA13* is the cause of all the phenotypic variations in *T16* (Fig. 5; Supplementary Fig. S5). In the fine-mapping work, we selected only plants with wilted leaves at the five-leaf stage for the identification of recombination events. Then, the plant height of these hypothesized recombinant individuals during the mature stage was examined; only dwarf plants similar to *T16* were regarded as true recombinant plants. Furthermore, these recombinant plants were self-pollinated and used to test the authenticity of the phenotype. This phenotype identification strategy reduces the enormous workload and uncertainty caused by the direct observation of vascular tissue and LRs and maximizes the accuracy of map-based cloning.

### Molecular mechanism of *BnaA03.IAA13* involvement in regulating vascular tissue and LR development

A typical Aux/IAA protein contains four complete domains. Domain I recruits the co-suppressor TPL to inhibit ARF activity (Szemenyei *et al*., 2008). Domains III and IV form heterodimers with ARF (Korasick *et al*., 2014). At the optimal auxin concentration, Aux/IAA interacts with TIR1 through domain II and degrades Aux/IAA proteins through ubiquitination, thereby releasing the transcriptional activity of ARF (Gray *et al*., 2001; Dreher *et al*., 2006; Tan *et al*., 2007; Figueiredo *et al*., 2022); this represents the classic auxin signaling pathway. In this study, *BnaA03.IAA13* was identified as encoding a typical Aux/IAA protein containing four complete domains (Fig. 5B), confirming that the product of *BnaA03.IAA13* can interact with TIR1 (Fig. 7E), indicating that *BnaA03.IAA13* participates in the classic auxin signaling pathway and regulates downstream signaling through the IAA13–ARFs module.

Auxin and cytokinin are essential for plant development (Bishopp *et al*., 2011; Sun *et al*., 2023) and complex crosstalk regulatory networks (Dello *et al*., 2008; Schlereth *et al*., 2010; Marhavý *et al*., 2011; Šimášková *et al*., 2015; Yang *et al*., 2017). Although the signal transduction mechanism of auxin and cytokinin is known, it remains unclear how plants integrate these two hormonal signals to regulate their growth. In this study, transcriptome analysis of the roots and stems showed differential expression of genes related to the auxin and cytokinin pathways (Fig. 7C, D; Supplementary Tables S4, S5), indicating that *BnaA03.IAA13* may integrate these two hormone pathways. CKXs degrade cytokinin irreversibly to maintain cytokinin homeostasis (Kowalska *et al*., 2010). *ARF5* inhibits cytokinin signaling in the primordial initiation region by directly activating *CKX6*, allowing lateral organs to initiate normally (Dai *et al*., 2023). *OsARF25* directly regulates *OsCKX4* to control root development in rice (Gao *et al*., 2014). The transcriptome and qRT–PCR results showed that *CKX1*, *CKX5*, and *CKX7* were down-regulated (Supplementary Fig. S9C-F). Multiple AuxREs exist in the promoter regions of CKXs, and expression pattern analysis showed they were expressed in all tissues (Supplementary Fig. S11). Hormone analysis indicated that there was a significant accumulation of cytokinin in the mutant (Fig. 7F, G). These results indicate that CKXs may be directly regulated by the IAA13–ARFs module, thereby affecting cytokinin homeostasis.

**Fig. 9.**
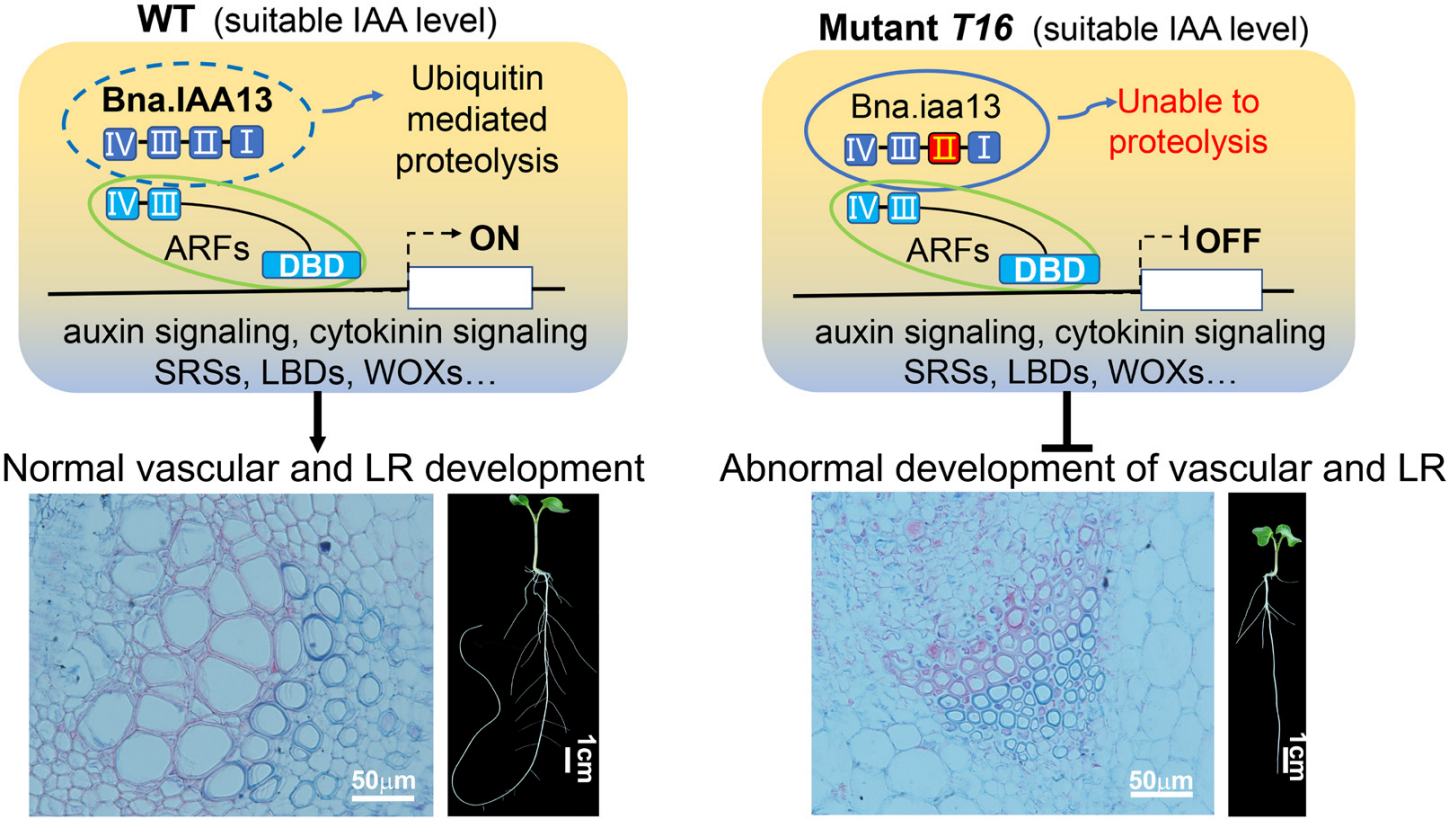
Model showing the involvement of BnaA03.IAA13 in regulating vascular and lateral root development in rapeseed. BnaA03.IAA13 inhibits the transcriptional regulatory activity of ARFs by interacting with them. When the concentration of auxin is appropriate, BnaA03.IAA13 undergoes ubiquitination degradation, releasing the transcriptional regulatory activity of ARFs. In the stem, ARFs affect plant growth and vascular development by regulating auxin and cytokinin signaling. In the roots, ARFs affect lateral root development through multiple pathways, such as SRSs, LBDs, WOXs, auxin, and cytokinin signaling.

Based on these results, we have proposed a model of *BnaA03.IAA13* participating in the development of vascular tissue and LR (Fig. 9). At optimal auxin concentrations, the protein product of *BnaA03.IAA13* interacts with TIR1 and is degraded, thereby releasing the regulatory activity of ARFs. The mutation in *BnaA03.IAA13* contributes to its inability to interact with TIR1 (Fig. 7E), resulting in the regulatory activity of ARFs being continuously inhibited, leading to the differential expression of auxin early-response genes (encoding Aux/IAA, GH3, and SAUR), causing disorders of the auxin signaling pathway. Furthermore, the mutation affects the regulation of *CKX5* by ARFs, leading to an imbalance in cytokinin homeostasis, which in turn affects the cytokinin signaling pathway, ultimately leading to dwarfism and abnormal vascular tissue development (Fig. 9). Similarly, auxin and cytokinin signals in the roots are disrupted, resulting in differential expression of genes related to LR development, such as *SRSs*, *LBDs*, and *WOXs*, which are directly regulated by ARFs (Suer *et al*., 2011; Brackmann *et al*., 2018; Singh *et al*., 2020; Yuan *et al*., 2020; Zhang *et al*., 2023). Together, these factors lead to abnormal root development (Fig. 9).

### The VGWPP motif is crucial for plant growth, and the Aux/IAA protein is evolutionarily conserved

The VGWPP motif interacts with TIR1/AFB to mediate Aux/IAA protein degradation, thus affecting the stability of Aux/IAA proteins and continuously inhibiting ARF activity (Gray *et al*., 2001; Dreher *et al*., 2006; Figueiredo *et al*., 2022). Mutation of this motif can cause developmental defects, which have been reported in many plants (Fukaki *et al*., 2002; Jun *et al*., 2011; Von *et al*., 2011; Su *et al*., 2022). In rapeseed, *BnaA03.iaa7* (V**E**WPP) causes dwarfism in the mutant *sca* and a decrease in the branch angle (Li *et al*., 2019). Interestingly, the mutation of the same gene at different positions (VGWP**L**) leads to the increased branching angle of the mutant *sdA03*, leaf crimpling, and root elongation (Ping *et al*., 2022). This observation indicates that mutations in the same Aux/IAA gene at different sites of the VGWPP motif may lead to different phenotypes, which may be related to the affinity of TIR1 (Li *et al*., 2019). Mutations of *BnaC05.IAA7* (VGW**L**P) result in leaf shrinkage and a reduction of LRs in the mutant *ed1* (Zheng *et al*., 2019). Mutations of *BnaC05.IAA2* (VGWP**S**) led to upward curling of the leaf blade of the mutant *NJAU5737* (Huang *et al*., 2020). In this study, the motif in *BnaA03.IAA13* was mutated to V**E**WPP, resulting in dwarfism, fewer LRs, and impaired development of vascular tissue. This indicates that different Aux/IAA gene mutations may lead to different phenotypes, suggesting that the Aux/IAA gene family is involved in regulating the development of different plant organs. Therefore, it is feasible to create different mutants by site-directed mutagenesis of the VGWPP motif, which could reveal additional functions of Aux/IAA proteins involved in regulating plant growth and development.

Typical Aux/IAA proteins contain four domains. Whole-genome Aux/IAA protein identification was carried out for algae (*Volvox carteri*, *Chlamydomonas reinhardtii*, and *Chondrus crispus*), bryophytes (*Physcomitrium patens* and *Marchantia polymorpha*), and pteridophytes (*Adiantum capillus-veneris* and *Selaginella moellendorffii*). The results showed that Aux/IAA exists in bryophytes and pteridophytes but not in algae. Moreover, the AUX/IAA proteins in bryophytes and pteridophytes have four complete domains similar to those in angiosperms, with the VGWPP motif being highly conserved (Supplementary Fig. S13). This research showed that *P. patens* can interact with the co-repressor complex TPL, and domain II can interact with TIR/AFB proteins (Prigge *et al*., 2010; Causier *et al*., 2012). In *M. polymorpha*, Aux/IAA can interact with ARF; transgenic lines expressing Aux/IAA containing a mutation in domain II showed an auxin-deficiency phenotype (Kato *et al*., 2015). These lines of evidence indicate that indicate that bryophytes share the same auxin response mechanism with angiosperms, the typical Aux/IAA originates from bryophytes, and its evolutionary process from bryophytes to angiosperms is conserved. Mutations in the Aux/IAA conservative domain during evolution, especially in the VGWPP motif, may cause serious growth defects and will be eliminated.

### The mutant gene *BnaA03.iaa13*, as a dose-sensitive gene, does not affect the number of seeds per silique or thousand-seed weight

The phenotype and qRT–PCR analysis of transgenic lines indicated a positive correlation between the degree of phenotypic variation and expression abundance of the mutated gene *BnaA03.iaa13* (Fig. 5D–I). The F_1_ hybrid showed a slight decrease in plant height and the number of LRs (Fig. 8; Supplementary Fig. S12), indicating that the mutated gene *BnaA03.iaa13* is a dose-sensitive gene. Yield analysis showed that the number of siliques per plant was significantly reduced in the mutant *T16* compared with the WT, which may be due to severe developmental defects in the root and stem. Interestingly, no difference in the number of seeds per silique and thousand-seed weight was observed between *T16* and WT plants, which may be because the nutrients for seed development in rapeseed originate from photosynthesis in the silique skin. In addition, although the F_1_ hybrid showed a slight decrease in plant height and LRs, no differences were observed in the number of siliques per plant, seeds per silique, thousand-seed weight, or yield per plant compared with the WT. Expression pattern analysis showed that *BnaA03.IAA13* was mainly expressed at a high level in roots and stems. These results indicate that *BnaA03.IAA13* mainly regulates the development of roots and stems and has an insignificant effect on the yield, suggesting that the mechanism of auxin signaling in regulating yield components and root and stem development differs.

Due to the long-term application of heterosis, the plant height of rapeseed varieties in China generally exceeds 180 cm, which makes rapeseed prone to lodging and hinders the development of mechanization and light-simplified cultivation. Therefore, the cultivation of dwarf varieties is significant to the Chinese rapeseed industry. The results of previous studies showed that regulating the expression of dose-sensitive genes to optimize crop yield is a feasible approach (Park *et al*., 2014; Je *et al*., 2016; Zhang *et al*., 2017; Hu *et al*., 2019), for example, how the weak allele of maize *fea3* increases maize yield (Je *et al*., 2016) and how fine-tuning the expression of IPA1 affects rice yield (Zhang *et al*., 2017). The results of the present study show that the F_1_ plant height (~130 cm) is suitable for rapeseed production. Most importantly, the yield of the F_1_ plants did not decrease relative to the WT (Fig. 8). Therefore, regulating the expression of *BnaA03.iaa13* to affect rapeseed development is a feasible strategy. The balance between the expression level of *BnaA03.iaa13* and yield warrants further studies.

## Supplementary data

The following supplementary data are available at *JXB* online.

Video S1. Leaf wilting process under high temperature (32 °C); original movie duration of 18 min, sped up to 10 s.

Video S2. Withered leaves in the greenhouse (20 °C) gradually returning to normal; original movie duration of 19 min, sped up to 10 s.

Fig. S1. SEM observation of stoma.

Fig. S2. Ultrastructural observation of the two largest vascular bundles in the petiole.

Fig. S3. Images of representative types of important recombinant plants after self-pollination.

Fig. S4. Sequence variation of *BnaA03.iaa13* in the mutant.

Fig. S5. Phenotypic characteristics of *BnaA03.iaa13* transgenic lines.

Fig. S6. qRT–PCR analysis of *BnaA03.iaa13* expression levels in transgenic lines.

Fig. S7. Comparison of IAA13 proteins in different species.

Fig. S8. qRT–PCR analysis of *BnaA03.IAA13* expression levels in different tissues.

Fig. S9. qRT–PCR analysis validation of transcriptome data.

Fig. S10. Transcriptome analysis of the *BnaA03.iaa13* transgenic lines.

Fig. S11. Analysis of CKXs promoter elements and expression patterns.

Fig. S12 Characterization of *T16* heterozygotes at the seedling stage.

Fig. S13. Alignments of four domains of AUX/IAA orthologs in bryophytes and pteridophytes.

Table S1. Primers used in this study.

Table S2. Differential SNPs information using *Brassica* 50 K SNP BeadChip Array analysis.

Table S3. Candidate gene analysis.

Table S4. Plant hormone signal transduction DEGs in stem transcriptome data.

Table S5. Plant hormone signal transduction DEGs in root transcriptome data.

Table S6. DEGs related to root development in the transcriptome.

Table S7. Key transcription factors are differentially expressed in the root transcriptome.

erae245_suppl_Supplementary_Figures_S1-S13

erae245_suppl_Supplementary_Tables_S1-S7

erae245_suppl_Supplementary_Video_S1

erae245_suppl_Supplementary_Video_S2

## Data Availability

The original datasets of RNA-seq for roots and stems are available in the NCBI Sequence Read Archive database (https://www.ncbi.nlm.nih.gov/sra; accession numbers PRJNA1013958, PRJNA1014376). All other relevant data can be found within the manuscript and its supplementary data online.
