# Correction to: A gain-of-function mutation in *BnaIAA13* disrupts vascular tissue and lateral root development in *Brassica napus*

**DOI:** 10.1093/jxb/erae345

**Published:** 2024-08-17

This is a correction to: Jinxiang Gao, Pei Qin, Shan Tang, Liang Guo, Cheng Dai, Jing Wen, Bin Yi, Chaozhi Ma, Jinxiong Shen, Tingdong Fu, Jun Zou, Jinxing Tu, A gain-of-function mutation in *BnaIAA13* disrupts vascular tissue and lateral root development in *Brassica napus*, *Journal of Experimental Botany*, 2024; erae245, https://doi.org/10.1093/jxb/erae245

In the originally published version of this manuscript, there was an error in the title, whereby “*BnaIAA13*” was given as “*BnAIAA13*”.

This has been corrected.

