# Supplementary material for: A gain-of-function mutation in BnaIAA13 disrupts vascular tissue and lateral root development in Brassica napus
Source: J Exp Bot. 2024 Jun 2;75(18):5592–610. doi: 10.1093/jxb/erae245 (PMC11427839; doi:10.1093/jxb/erae245)
Supplement: erae245_suppl_Supplementary_Figures_S1-S13 [file erae245_suppl_supplementary_figures_s1-s13.pdf]

Supplementary Figures

Fig. S1 SEM observation of stoma.

Fig. S2 Ultrastructural observation of the two largest vascular bundles in the petiole.

Fig. S3 Observation on representative types of important recombinant plants after self-pollination.

Fig. S4 Variation information of *BnaA03.iaa13* in the mutant.

Fig. S5 Phenotypic identification of *BnaA03.iaa13* transgenic lines.

Fig. S6 qRT-PCR analysis of *BnaA03.iaa13* expression levels in transgenic lines.

Fig. S7 Comparison of *IAA13* proteins in different species.

Fig. S8 qRT-PCR analysis of *BnaA03.IAA13* expression levels in different tissues.

Fig. S9 qRT-PCR analysis validation of transcriptome data.

Fig. S10 Transcriptome analysis of the *BnaA03.iaa13* transgenic lines.

Fig. S11 Analysis of CKXs promoter elements and expression patterns.

Fig. S12 Characterization of *T16* heterozygous at seedling.

Fig. S13 Alignments of four domains of AUX/IAA orthologs in bryophytes and pteridophytes.

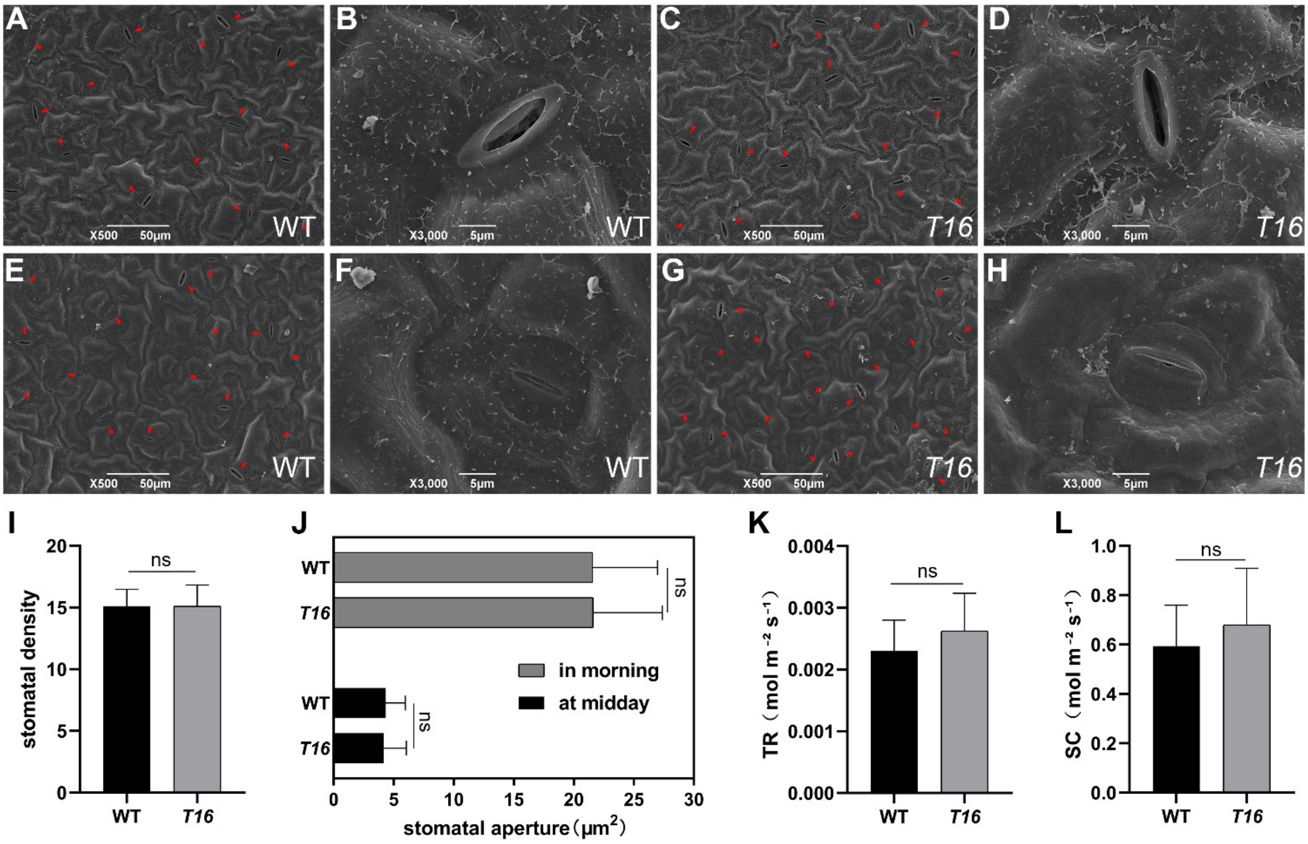

Fig. S1 SEM observation of stoma. (A-D) Observation of stomata at 9:00 AM. The red triangle indicates stomata. (E-H) Observation of stomata at 1:00 PM. The red triangle indicates stomata. (I and J) Statistical analysis of stomatal density and stomatal aperture. No significant difference. Values in column charts represent the mean ± SD of density and aperture of all stomatal cells in 10 views. (K and L) Measurement and analysis of transpiration rate and stomatal conductance. No significant difference. Using LI-6800 Photosynthesis Fluorescence Fully Automatic Detection System. Perform three biological replicates in SEM. Values in column charts represent the mean ± SD (n = 5). Statistically significant differences were revealed using Student’s t-test.

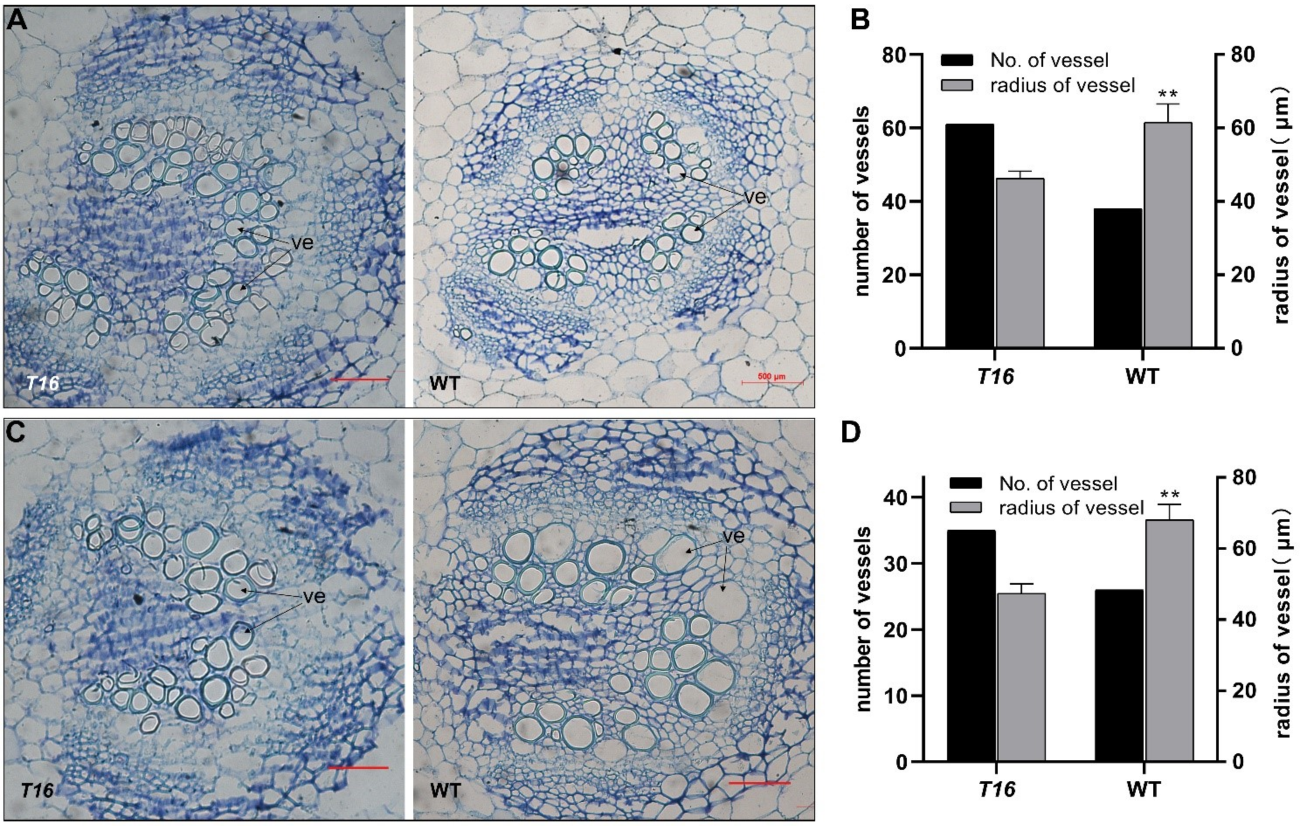

Fig. S2 Ultrastructural observation of the two largest vascular bundles in the petiole. (A, C) The largest vascular bundles in the petiole of *T16* and WT (bar = 500 μm). (B, D) Statistical analysis of the vessel number and vessel radius, measure the radius of the five largest conduits. ve: vessel. Values in column charts represent the mean ± SD (n = 5). Using Student's *t*-test: ** P <0.01.

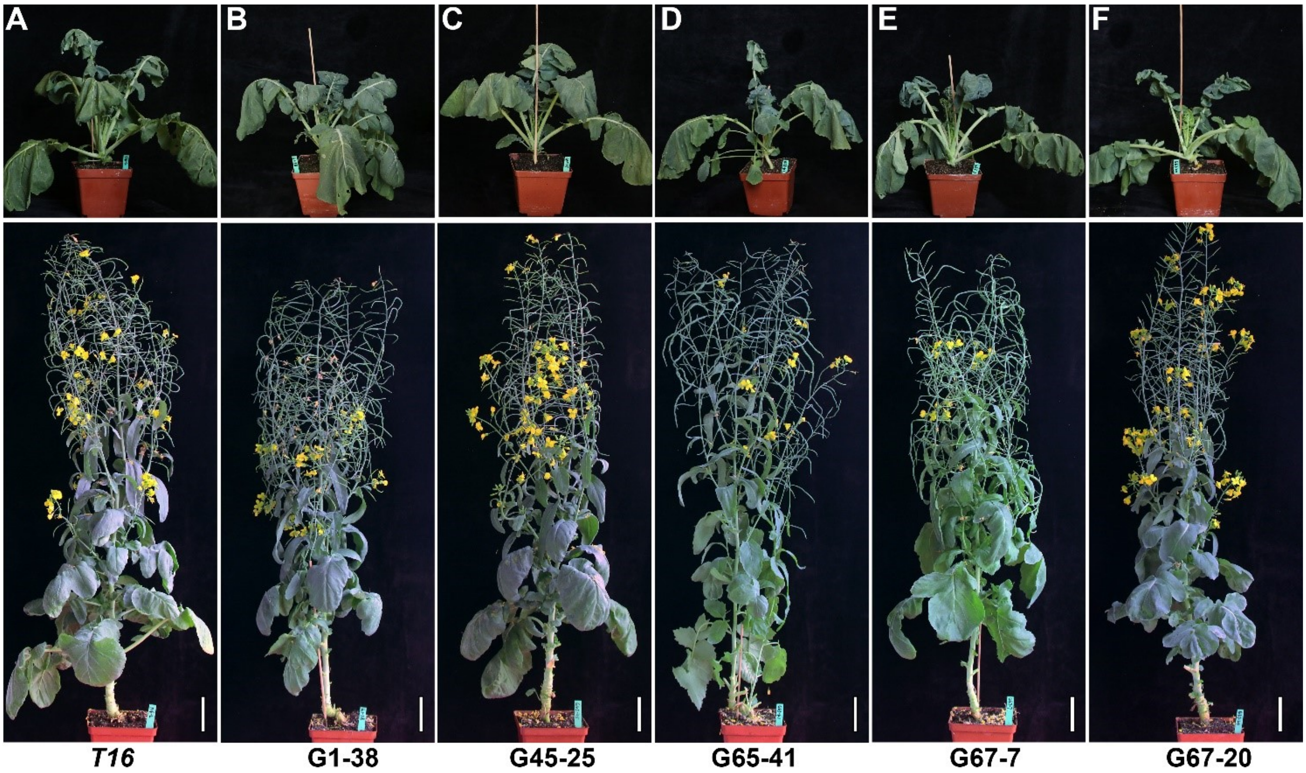

Fig. S3 Observation on representative types of important recombinant plants after self-pollination. Fine mapping 59.6 kb contains five recombinant plants, with selfing offspring exhibiting dwarfing and leaf wilting like *T16*, supporting the reliability of the fine mapping results (bar = 5 cm).

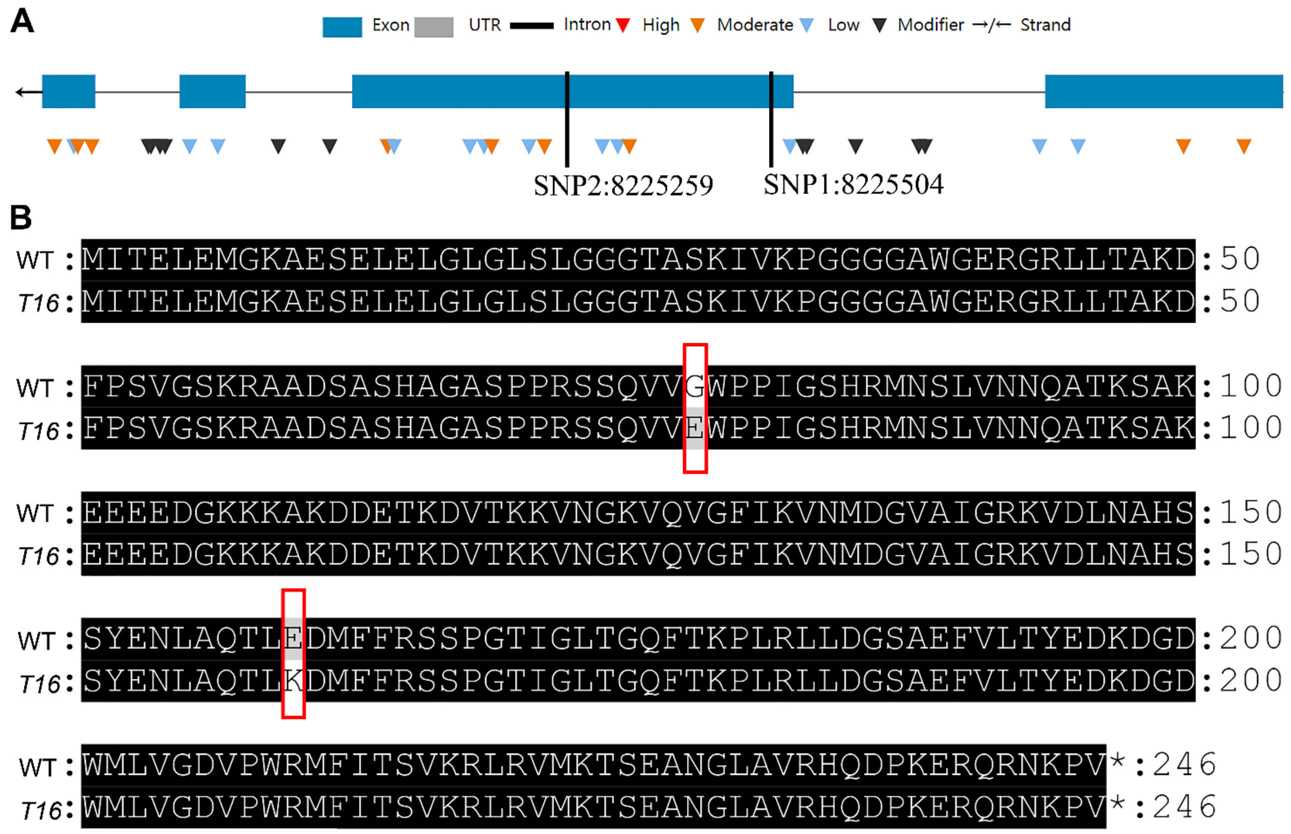

Fig. S4 Variation information of *BnaA03.IAA13* in the mutant. (A) Two SNPs variation information of *BnaA03.IAA13* and haplotype distribution in natural populations (Triangles represent different Haplotype, and different colors represent frequencies in natural populations). (B) Comparison of *BnaA03.IAA13* protein sequence between the WT and the *T16*. Red box represents the mutation site of the *T16*.

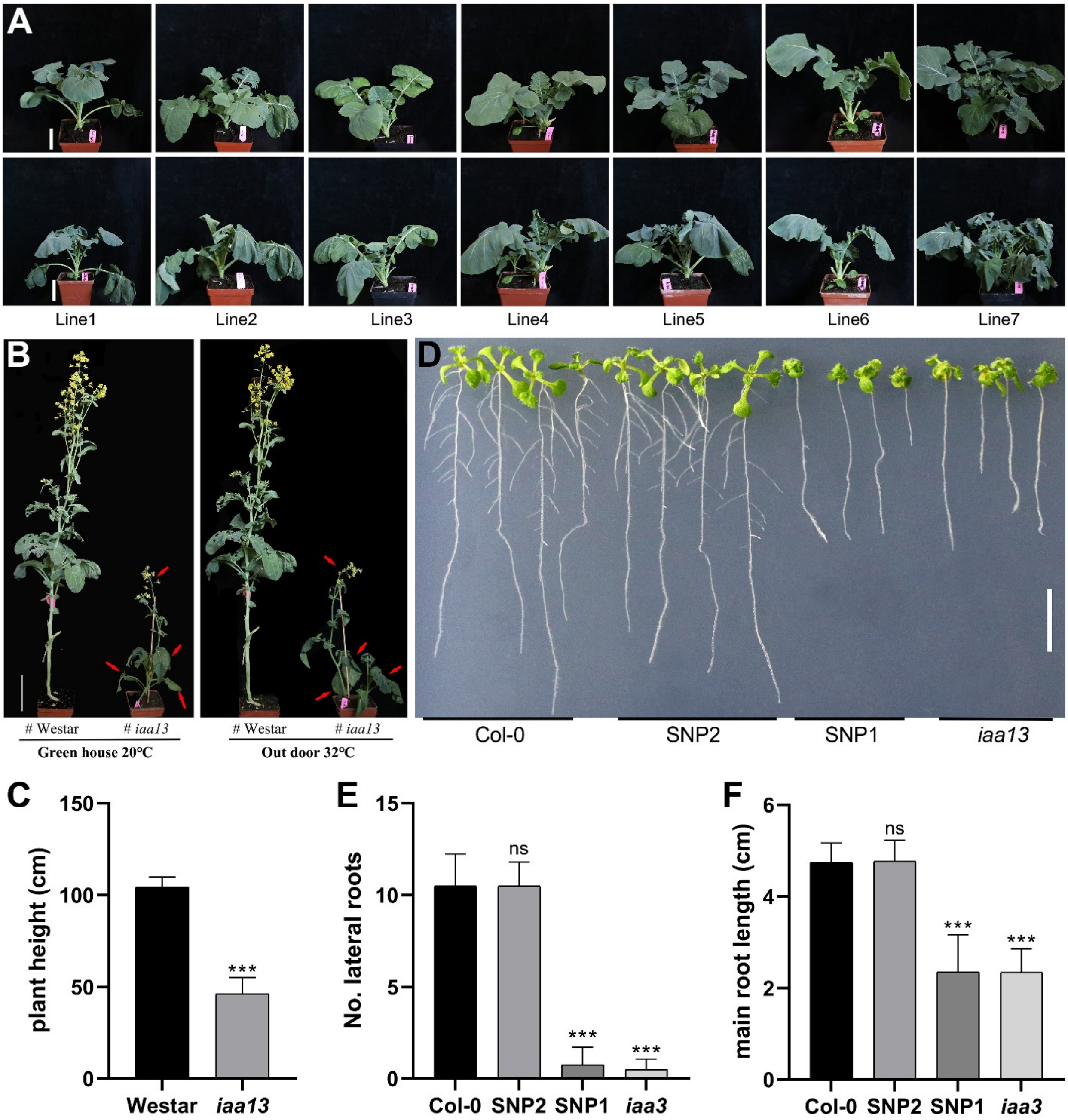

Fig. S5 Phenotypic identification of mutated gene *BnaA03.iaa13* transgenic lines. (A) Phenotype of mutated gene *BnaA03.iaa13* positive independent transgenic lines in normal material ZS11. First row is the phenotype in greenhouse, and the second row is the phenotype at 30 ℃ outdoors. The obvious wilting phenotype can be observed after transferring to the outside for about 5 minutes (bar = 5 cm). (B) Phenotypes of mutated gene *BnaA03.iaa13* positive independent transgenic plants in normal material Westar during the flowering stage, arrows indicate leaf wilting (bar = 10 cm). (C) Statistical analysis of plant height of *BnaA03.iaa13* transgenic plants in rapeseed. (D) Root phenotype of mutated gene *BnaA03.iaa13* positive independent transgenic plants in Arabidopsis (bar =1 cm). (E, F) Statistical analysis of lateral root number and main root length in Arabidopsis. Values in column charts represent the mean ± SD (n = 12). Statistically significant differences were revealed using Student's t-test: *** $P < 0.001$.

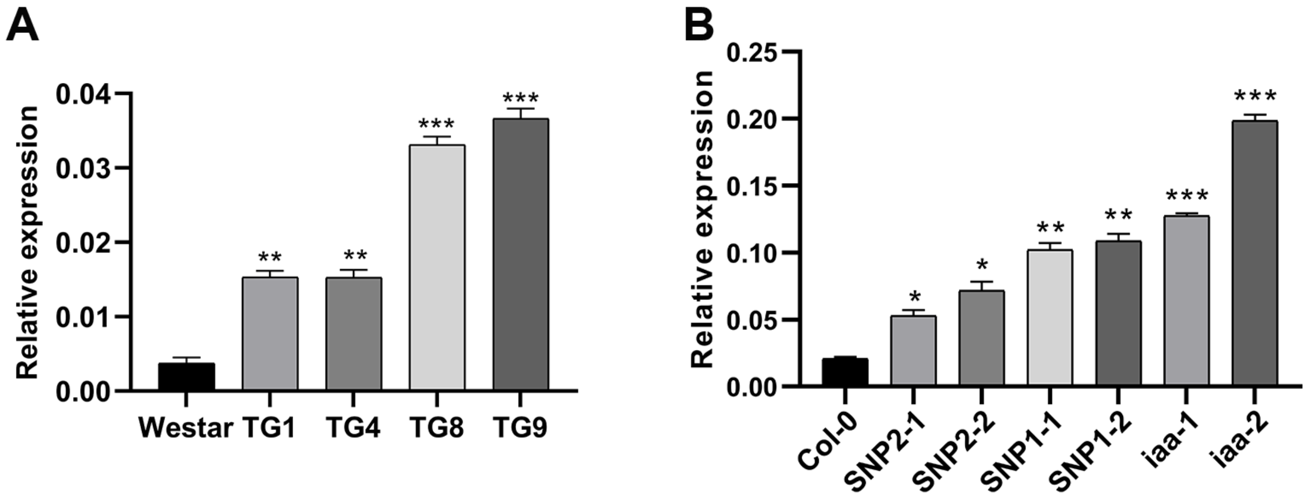

Fig. S6 qRT-PCR analysis of *BnaA03.IAA13* expression levels in transgenic lines. (A) qRT-PCR analysis in rapeseed transgenic lines by using *BnaENTH* as a reference gene. (B) qRT-PCR analysis in Arabidopsis transgenic lines by using *AtActin7* as a reference gene. The values in column charts represent the mean ± SD (n = 3). Using Student's *t*-test: * $P < 0.05$, ** $P < 0.01$, *** $P < 0.001$.

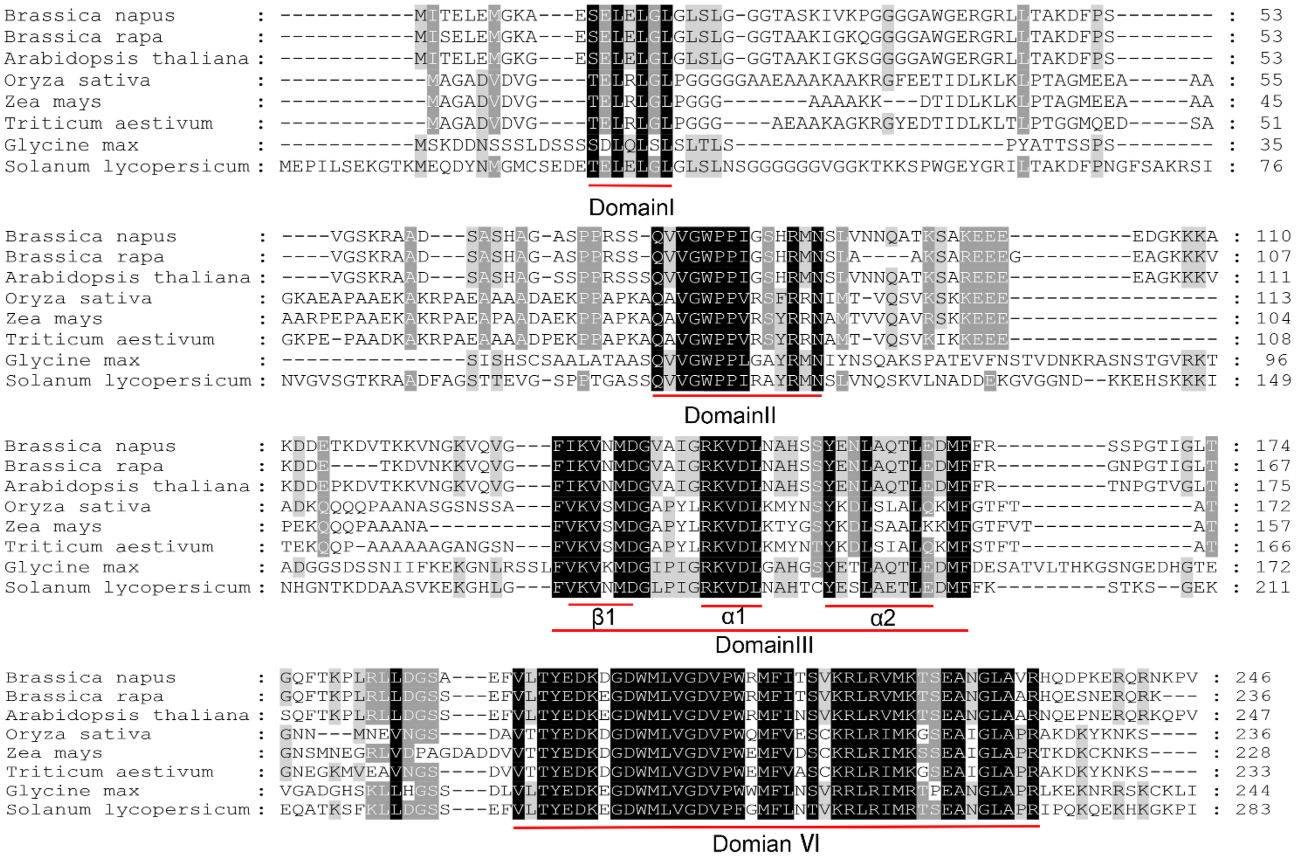

Fig. S7 Comparison of *IAA13* proteins in different species. *Brassica napus, Brassica rapa, Arabidopsis thaliana, Oryza sativa, Zea mays, Triticum aestivum, Glycine max, Solanum lycopersicum.* Download protein sequences from NCBI. Visualize using MEGA7 and GENEDOC.

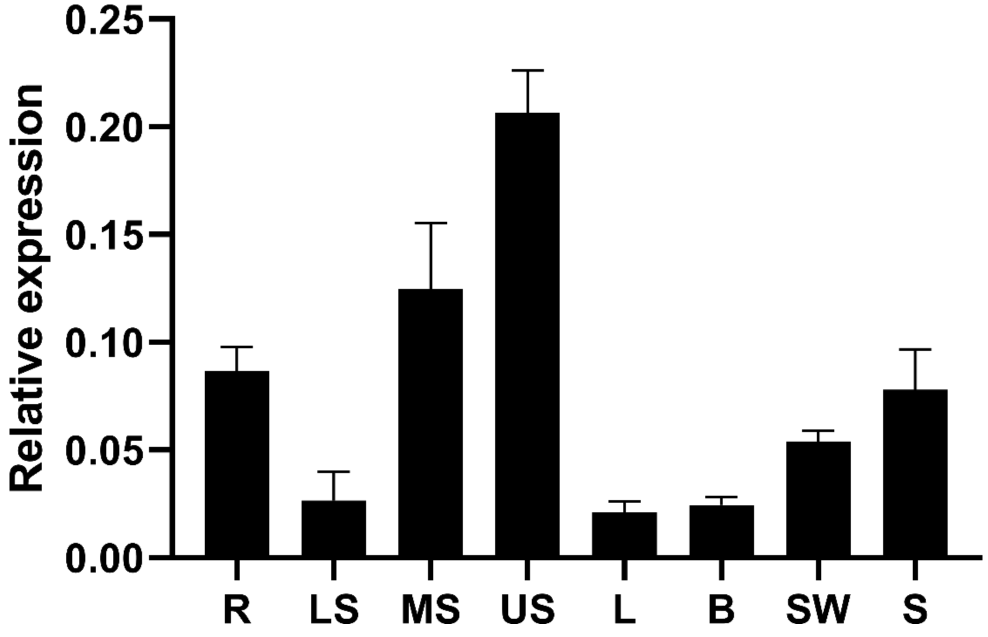

Fig. S8 qRT-PCR analysis of *BnaA03.IAA13* expression levels in different tissues by using *BnaENTH* as a reference gene. R: root; LS: low stem; MS: middle stem; US: up stem; L: leaf; B: bud; SW: silique wall; S: seed. Values represent the mean ± SD (n = 3).

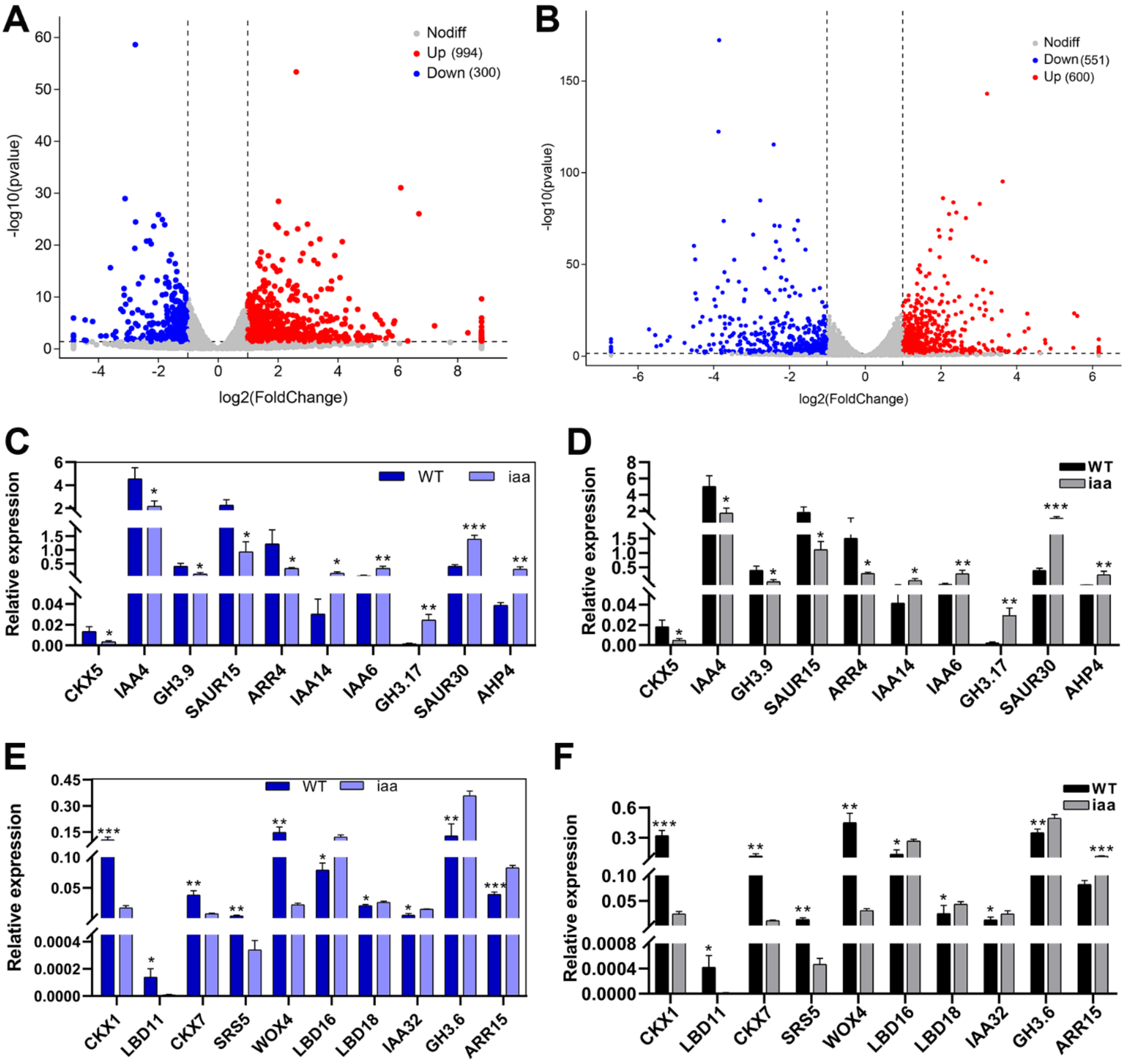

Fig. S9 qRT-PCR analysis validation of transcriptome data. (A) Volcano plot of stem transcriptome analysis. (B) Volcano plot of root transcriptome analysis. (C) qRT-PCR analysis validation of stem transcriptome results by using *AtActin2* as a reference gene. (D) qRT-PCR analysis validation of stem transcriptome results by using *AtActin7* as a reference gene. (E) qRT-PCR analysis validation of root transcriptome results by using *AtActin2* as a reference gene. (F) qRT-PCR analysis validation of root transcriptome results by using *AtActin7* as a reference gene. RNA used in RNA-seq was applied to test the transcriptome data by qRT-PCR. Values in column charts represent the mean ± SD (n = 3). Using Student's *t*-test: * $P < 0.05$, ** $P < 0.01$, *** $P < 0.001$.

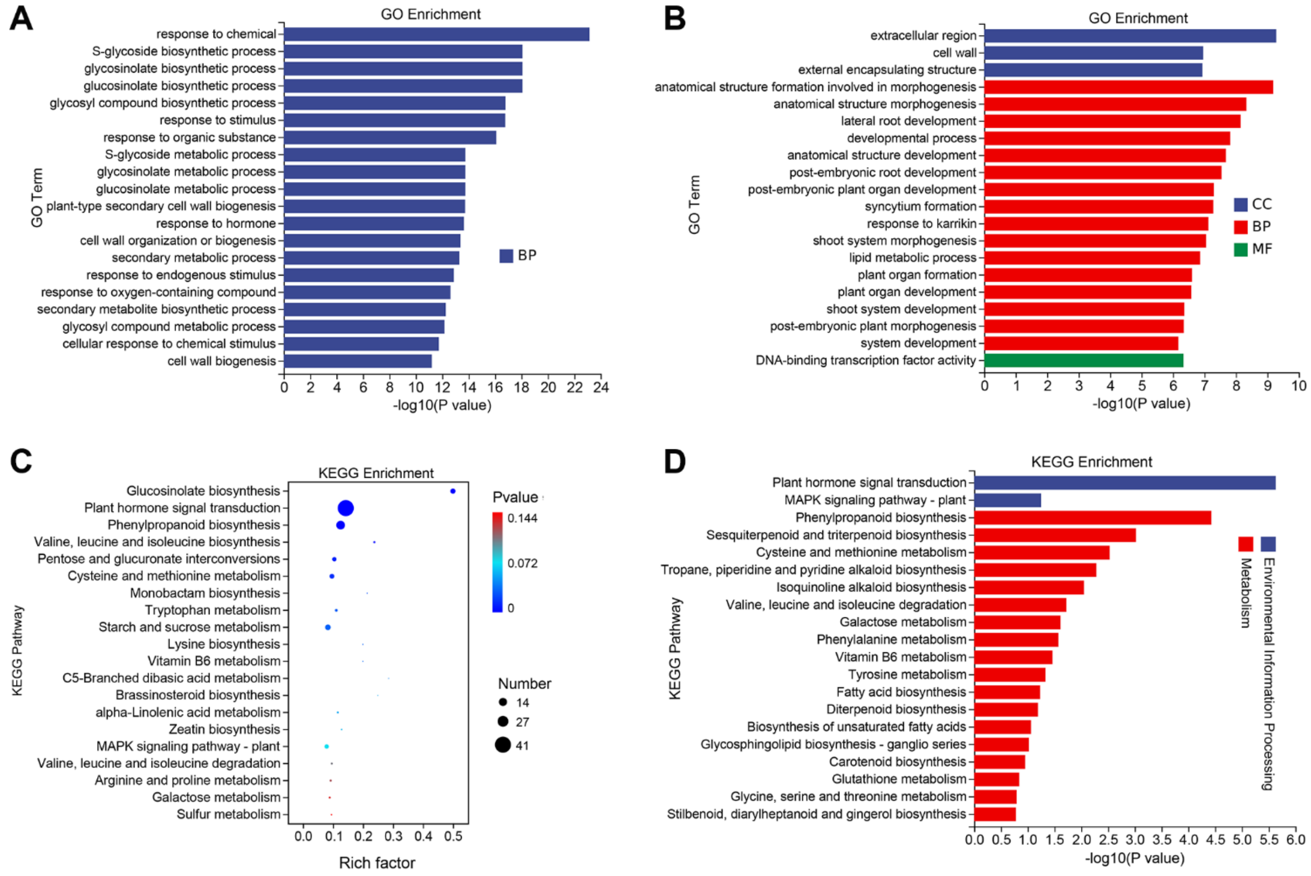

Fig. S10 Transcriptome analysis of the *BnaA03.iaa13* transgenic lines. (A) GO enrichment analysis of differentially expressed genes (DEGs) in stem RNA-seq. (B) GO enrichment analysis of DEGs in root RNA-seq. (C) KEGG enrichment analysis of DEGs in stem RNA-seq. (D) KEGG enrichment analysis of DEGs in root RNA-seq.

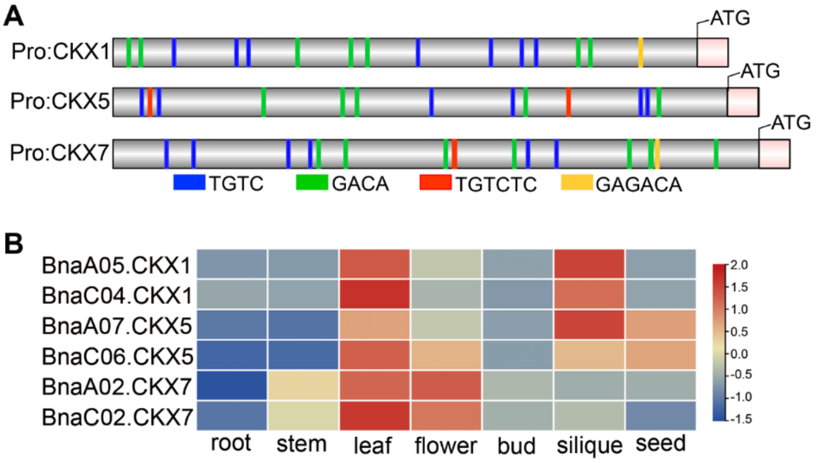

Fig S11 Analysis of CKXs promoter elements and expression patterns. (A) Analysis of AuxRE elements in the promoter region of CKXs. Download the 2,000 bp assumed promoter region of CKX from BnIR (https://yanglab.hzau.edu.cn/BnIR), analyze the AuxRE element online using PlantCARE (https://bioinformatics.psb.ugent.be/webtools/plantcare/html/), and visualize using IBS. (B) Expression levels of CKX1, CKX5, and CKX7 in various tissues of rapeseed. Download CKXs expression data from BnIR and visualize using TBtools.

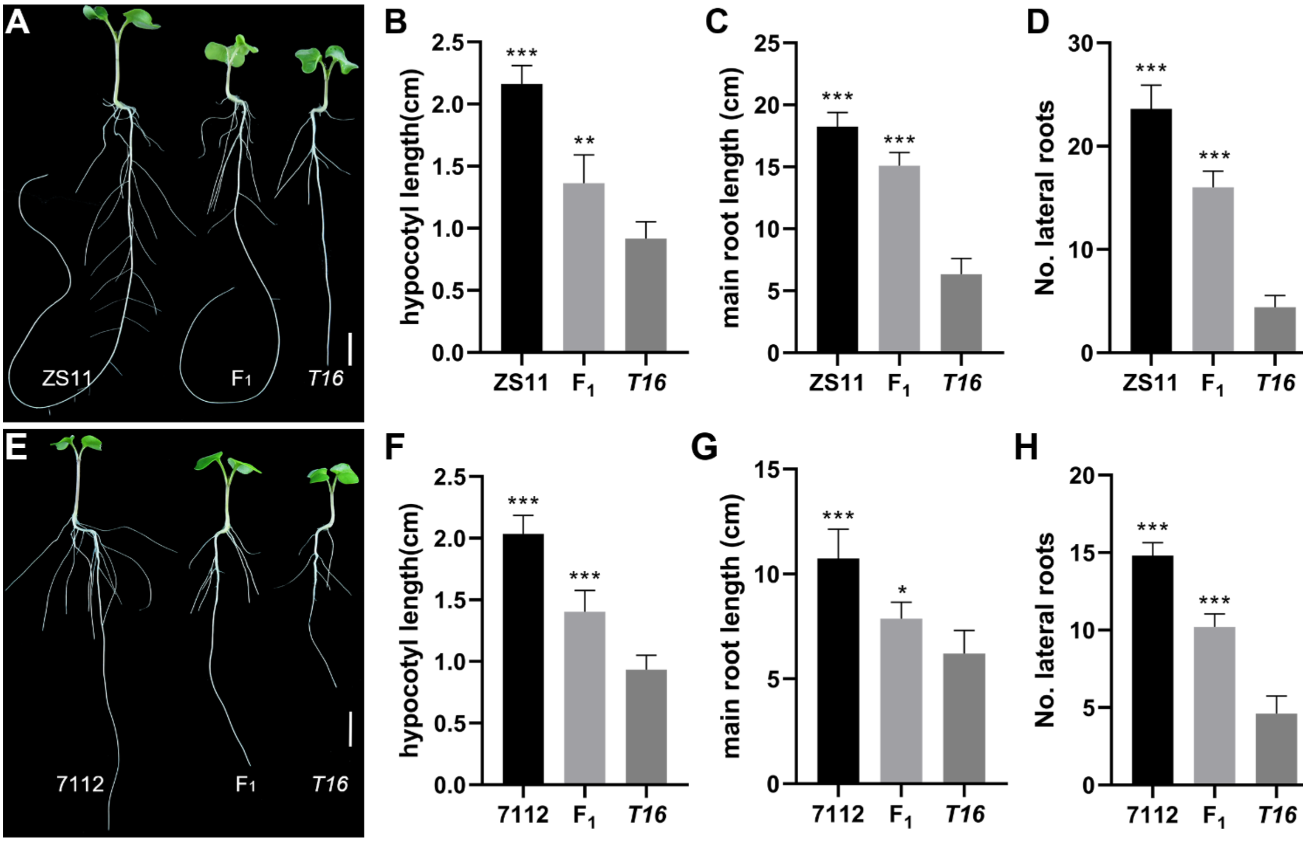

Fig. S12 Characterization of *T16* heterozygous at seedling. (A) Phenotype of *T16* and excellent variety ZS11 hybrids (bar = 1 cm). (B-D) Statistical analysis of hypocotyl length, main root length, and lateral roots number. (E) Phenotype of *T16* and inbred line 7112 hybrids (bar = 1 cm). (F-H) Statistical analysis of hypocotyl length, main root length, and lateral roots number. Values in column charts represent the mean ± SD (n = 10). Using Student's t-test: * $P < 0.05$; ** $P < 0.01$; *** $P < 0.001$.

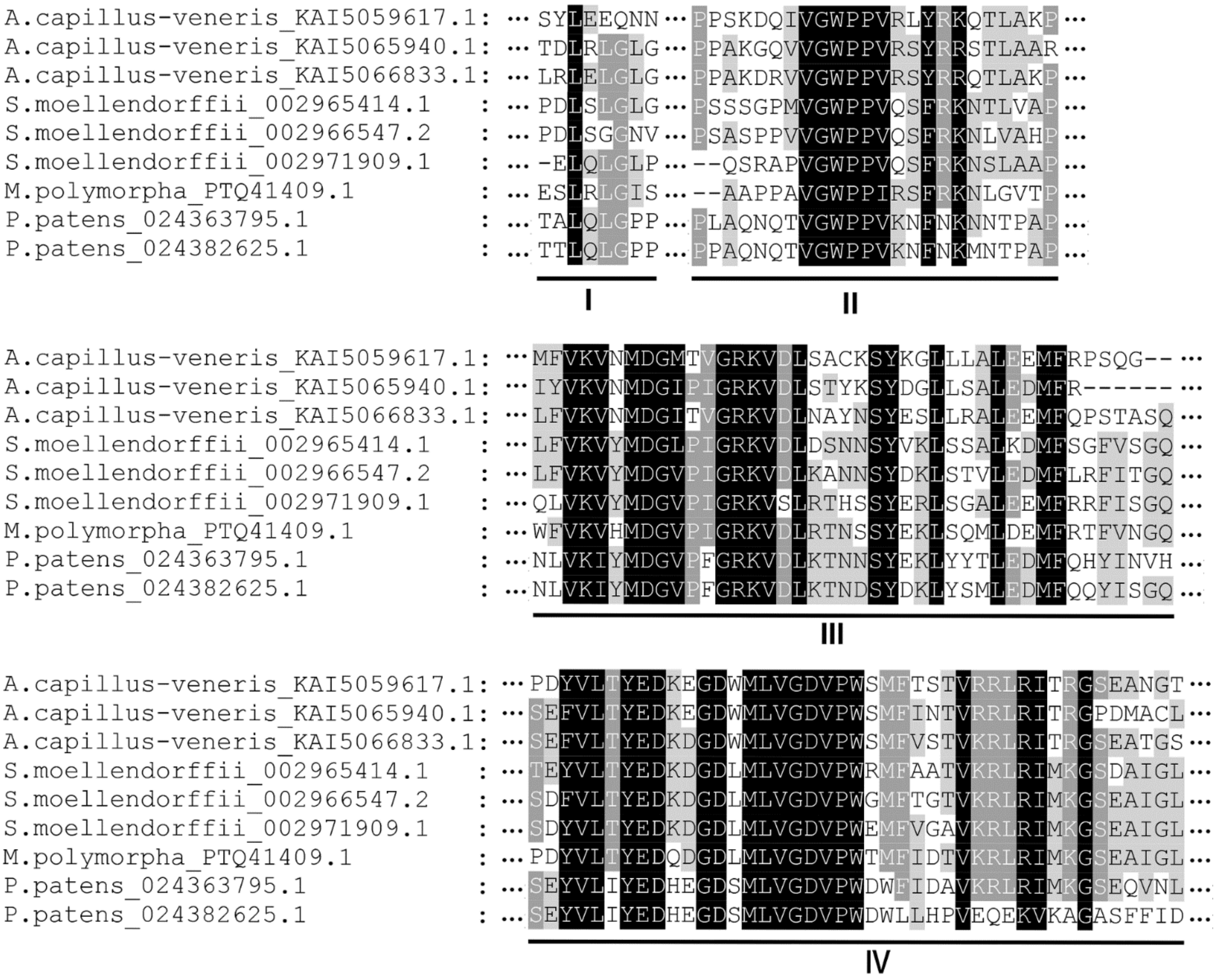

Fig. S13 Alignments of four domains of AUX/IAA orthologs in bryophytes and pteridophytes. AUX/IAA proteins in bryophytes and pteridophytes have four complete domains similar to those in angiosperms, with VGWPP motif being highly conserved. Download protein sequences from NCBI. Visualize using MEGA7 and GENEDOC.
